# Is age at menopause decreasing? – The consequences of not completing the generational cohort

**DOI:** 10.1186/s12874-022-01658-x

**Published:** 2022-07-11

**Authors:** Rui Martins, Bruno de Sousa, Thomas Kneib, Maike Hohberg, Nadja Klein, Elisa Duarte, Vítor Rodrigues

**Affiliations:** 1 Departamento de Estatística e Investigação Operacional, Faculdade de Ciências, Universidade de Lisboa, Portugal; Centro de Estatística e Aplicações da Universidade de Lisboa (CEAUL), Lisboa, Portugal; 2 Faculty of Psychology and Education Sciences (FPCE); Center for Research in Neuropsychology and Cognitive and Behavioral Intervention (CINEICC), University of Coimbra, Coimbra, Portugal; 3 University of Goettingen, Chair of Statistics, Humboldtallee 3, Goettingen, 37073 Germany; 4 Humboldt-Universität zu Berlin, School of Bus. Econ., Applied Statistics, Unter den Linden 6, Berlin, 10099 Germany; 5 Faculty of Medicine, University of Coimbra, Rua Larga, Coimbra, 3004-504 Portugal; 6 Liga Portuguesa Contra o Cancro, Núcleo Regional do Centro, Rua Dr. António José de Almeida, 329 - piso 2 - Sala 56, Coimbra, Portugal

**Keywords:** Copula function, Distributional regression, GJRM, Incomplete data, Menopause, Smoothing

## Abstract

**Background:**

Due to contradictory results in current research, whether age at menopause is increasing or decreasing in Western countries remains an open question, yet worth studying as later ages at menopause are likely to be related to an increased risk of breast cancer. Using data from breast cancer screening programs to study the temporal trend of age at menopause is difficult since especially younger women in the same generational cohort have often not yet reached menopause. Deleting these younger women in a breast cancer risk analyses may bias the results. The aim of this study is therefore to recover missing menopause ages as a covariate by comparing methods for handling missing data. Additionally, the study makes a contribution to understanding the evolution of age at menopause for several generations born in Portugal between 1920 and 1970.

**Methods:**

Data from a breast cancer screening program in Portugal including 278,282 women aged 45–69 and collected between 1990 and 2010 are used to compare two approaches of imputing age at menopause: (i) a multiple imputation methodology based on a truncated distribution but ignoring the mechanism of missingness; (ii) a copula-based multiple imputation method that simultaneously handles the age at menopause and the missing mechanism. The linear predictors considered in both cases have a semiparametric additive structure accommodating linear and non-linear effects defined via splines or Markov random fields smoothers in the case of spatial variables.

**Results:**

Both imputation methods unveiled an increasing trend of age at menopause when viewed as a function of the birth year for the youngest generation. This trend is hidden if we model only women with an observed age at menopause.

**Conclusion:**

When studying age at menopause, missing ages must be recovered with an adequate procedure for incomplete data. Imputing these missing ages avoids excluding the younger generation cohort of the screening program in breast cancer risk analyses and hence reduces the bias stemming from this exclusion. In addition, imputing the not yet observed ages of menopause for mostly younger women is also crucial when studying the time trend of age at menopause otherwise the analysis will be biased.

**Supplementary Information:**

The online version contains supplementary material available at (10.1186/s12874-022-01658-x).

## Introduction

Age at menopause has an important role in the research about risk factors for breast cancer [1]. However, it is a variable prone to incompleteness, because the time when women participate in a breast cancer screening program overlaps the time when women are most likely to enter menopause. Therefore, the younger women of the generation cohort under analysis tend to have missing information on age at menopause. Not recovering the values for age at menopause can lead to wrong conclusions because the parameter estimates for the most recent years will tend to be dominated by these young women.

Nowadays, there is greater awareness about discarding individuals with some missing observation from the statistical analysis. Generally, leaving out incompletely observed individuals tends to be unsatisfactory and unnaturally decreases the data sample. A simple imputation of the gaps using the mean of the respective variable leads to negative side effects as well since the covariance structure is neglected, i.e. set to zero, thus implying the variance estimators to be biased. Essentially, the literature handles incomplete data in two ways: (a) analysing only the cases with a complete vector of observations (complete cases analysis – CCA) and (b) analysing all the cases after imputing the missing observations with an appropriate statistical technique.

The question of whether missing values of a variable are related to the underlying value itself allows for classifying the missing data mechanism into three categories [2, 3]: missing completely at random (MCAR), missing at random (MAR) and missing not at random (MNAR). The data are said to be MCAR if the probability of a value being missing is neither related to the observed and unobserved values of that variable nor to other measured characteristics. In this scenario, the observed data are said to be representative of the overall data and analysing only the participants with a complete data vector is a valid approach. MAR is a less restrictive assumption, occurring when the probability of missing observations for a variable is related to other observed variables but unrelated with unobserved values given all other observed variables. The probability of a value being missing may be dependent on observed data but, given the observed data, is conditionally independent of the underlying value itself. This assumption means that outcomes for individuals with similar observed characteristics will have the same probability distribution, whether or not they have been observed. In this situation there exists a separation between the parameters of the missing process and the parameters of the observed response data – the missing process is said to be ignorable or non-informative. Data are MNAR if the probability of a value being missing is related to the values supposed to be observed for the variable at the time of the observation process – the missing process is said to be non-ignorable or informative. This implies that a missing observation has a different probability distribution than the observed values of other individuals even when they have the same characteristics. The validity of inferences made under different statistical methods depends on the assumption about the missing process. It is well known in these cases that inference-based statistical analysis ignoring such feature may lead to biased parameter estimates [3].

We frame the issue of imputing age at menopause as a missing data problem since we consider age at menopause as a covariate in a potential subsequent risk cancer analysis. We therefore ask the same question as in a classical missing value setting: Is the missing mechanism informative or not? Note that recovering the values for age at menopause as the dependent variable could also be treated as a censoring or prediction problem but is not the focus of this work.

To test how different strategies to impute missing ages at menopause for the youngest women influence the analysis of time- and spatial-trends of that variable, we will analyse the case of a breast cancer screening program in central Portugal. Exploratory analyses show the presence of a geographical pattern of the missing data and a close relation with a woman’s year of birth, implying, at least, a violation of the missing complete at random assumption. Additionally, there is a high percentage of missing values in the variable menopause (23.6%), which precludes an analysis by simply deleting those individuals.

Regarding time and spatial trends of age at menopause, recent researches have shown some contradictory conclusions. For instance, Duarte et al. [4] in a complete cases scenario stated that women born after the first world war are having their menopause at lower ages. On the other hand Dratva et al. [5] claim that there is a shift towards higher ages. Concerning the spatial patterns in the breast cancer’s relative risk for the central region in Portugal, there are also different findings. Rodrigues [6] reported a non-homogeneous risk across the municipalities, but Duarte et al. [7] reported a non-significant spatial effect.

To achieve the goals defined above, we will consider two statistical modelling approaches with the aid of two R packages, namely GJRM (v. 0.2-3) – Generalised Joint Regression Modelling [8] and gamlss (v. 5.1-7) – Generalised Additive Models for Location, Scale and Shape [9]. The GJRM package allows us to deal simultaneously with two response variables while their specific marginal distributions are conveniently expressed in a joint manner by means of a copula function that binds them together. In this way, we will be able to define a joint distribution for both the process that governs the probability that a woman has not yet reached menopause and for the age at menopause itself. A bivariate copula regression model will be adopted [10]. To allow for sufficient flexibility in the model estimation, we will consider spline functions to model some of the covariates effects. The gamlss package allows for virtually expressing any distributional parameter as a function of covariates in a generalized additive model (GAM, [11]) fashion and adopts a method for the imputations which is more flexible than other imputation methods provided by other packages in R [12]. This usage has naturally led to the emergence of a secondary objective of this work – to compare, within our context of age at menopause, the imputations obtained by these two different methods.

The remainder of this paper develops as follows: in “Breast cancer screening data from Portugal” section, we describe the motivating data set and present a brief exploratory analysis followed by a recall of some key definitions from the copulas literature (“Bivariate conditional copula regression” section). Two different imputation approaches are presented in “Imputation methodology” section, whereas “Modelling the age at menopause in central Portugal” section outlines and formalizes the main models. In “Results” section, we conduct a data analysis by applying a selected model chosen from a set of several similar models, present and discuss the results of the models. A sensitivity and validation analysis are presented in the Supplementary files. Concluding remarks and discussion of important related issues are given in “Discussion” section.

## Breast cancer screening data from Portugal

**Fig. 1:**
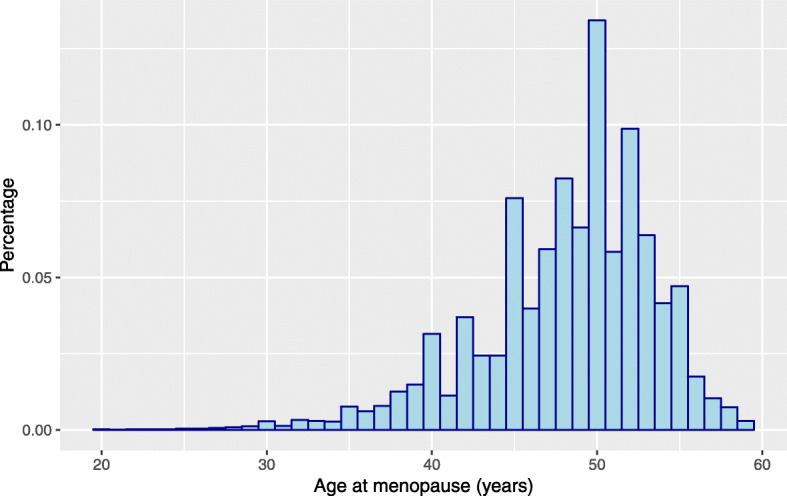
Age at menopause for the women with an observed value

**Fig. 2:**
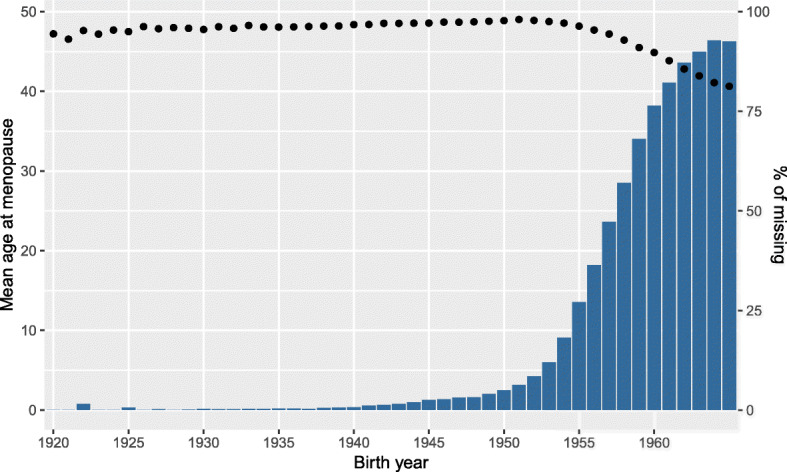
Histogram of the missing ages at menopause by birth year and points representing the mean age at menopause by birth year

**Fig. 3:**
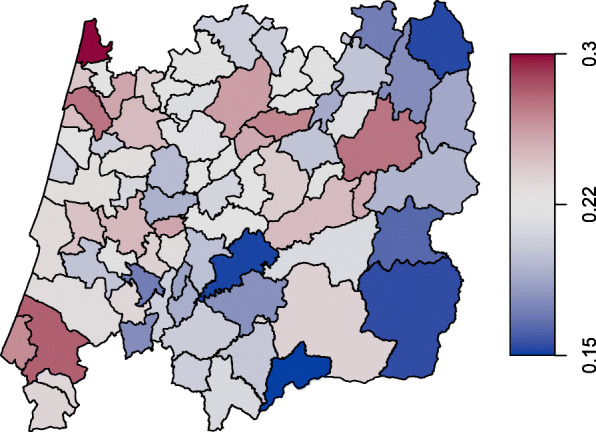
Missing ages at menopause by municipality

**Fig. 4:**
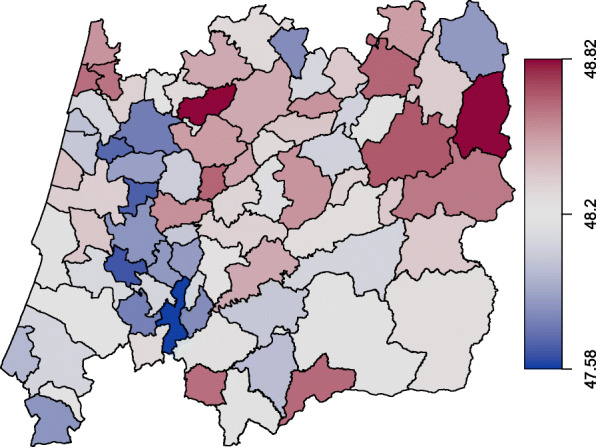
Mean age at menopause by municipality

The database that we are working with is constantly updated with longitudinal information from new women and from women who are already part of it. The records have the follow-up of 278 282 women between 1990 and 2010. At the age of 45 (since 2017 the onset age is 50), all women in each of the 78 municipalities are invited to have a free screening mammogram and every two years thereafter until the age of 69. At the time of the last screening, 65 765 women (23.6%) stated they had not yet reached menopause (missing information). Table 1 sum- marizes the variables included in the data set, namely (i) binary characteristics provided by the variables pregnancy (pregnancy=0 if the woman has never been pregnant; 1 otherwise), breastfeeding (breastf=0 if the woman has never breastfed; 1 otherwise) and the use of oral contraceptives (anov=0 if the woman has never used oral contraceptives; 1 otherwise); (ii) quantitative information carried by the continuous variables age at menopause (menopause) (Figs. 1 and 2), age at menarche (menarche), year of birth (birth) and age at the last attending screening (sage); (iii) demographic information given by the municipality purchasing power index (ipccap); and (iv) spatial information embodied in neighbourhood structure of the municipality of residence (muni). The central region of Portugal is divided in 78 municipalities (Figs. 3 and 4) and roughly represents 25% of the Portuguese population. More details about screening program and the inclusion criteria are given in [4].

**Table 1:** Summary of the continuous (top) and binary (bottom) variables used in data analysis

Variable	Summary	
	**Mean**	**Range**
Age at menopause	48.2	20–59
Age at menarche	13.2	8–18
Age at last attending screening	58.3	45–69
Year of birth	1948.9	1920–1965
Municipality purchasing power index	81	24–145
	**% No**	**% Yes**
Any pregnancy	7.4	92.6
Oral contraceptives	52.6	47.4
Breastfeeding	44.6	55.4

**Fig. 5:**
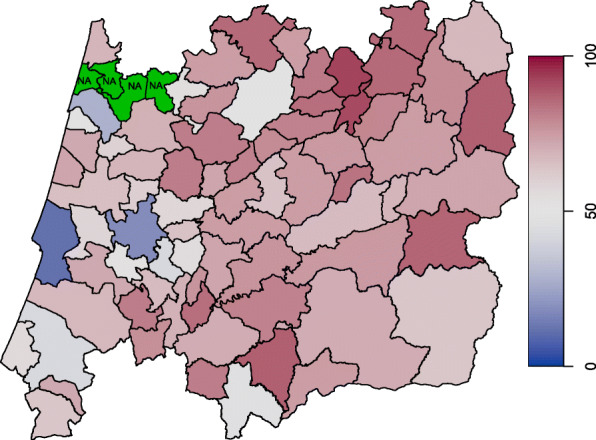
Participation rate by municipality. The NA represent information not available

To encourage participation in the screening program, invitation letters are sent out to women but the decision to participate is exclusively left to the women. In Fig. 5, the different levels of attendance per region are shown. Absenteeism is stronger in the coastal (Western) areas. The different reasons of non-attendance pointed out by many studies are unfavourable socio-economic levels, living in an urban region, or women that take care of their health by their own initiative [13, 14].

In 2017, we had been granted access to 20 130 women already screened in 2010 and who have since then reached menopause. With these data at hand, we can compare the imputed values for those women in 2010 with their real age at menopause allowing us to check the reliability of the obtained results under the assumed missing mechanism. This validation analysis, for the sake of space, is carried out in one additional file available online.

## Bivariate conditional copula regression

Although some ages at menopause, especially these of younger women, cannot be directly observed, we can retrieve some information based on the idea of constructing a bivariate joint distribution of the missing data mechanism and the age at menopause assuming the data are MNAR. This allows us to input the not yet observed ages of menopause in order to complete the data set. In what follows, we give a brief introduction to the concept of copula function that facilitates the construction of such joint distribution.

### Bivariate joint distributions through copulas

Copulas are multivariate distribution functions that can be used to construct a dependence structure between two or more variables. Irrespective of the nature of the marginal distributions, copulas allow to investigate this dependence by combining the margins into a multivariate structure, usually accomplished in two steps: (i) choosing the optimal margins and (ii) choosing the optimal copula [15]. With copulas, the marginal behaviour (marginal distribution functions) is separated from the dependence structure. Usually, if one starts from a multivariate distribution function to represent joint probabilities, separating the dependence from the marginals is not achievable.

A bivariate copula *C*(.) is a distribution on [0,1]^2^→[0,1] for any set of two random variables *Y*_1_ and *Y*_2_ with univariate marginal distributions $F_{Y_{k}}(y_{k}), k=1,2$. The construction

1 $$ F_{Y_{1},Y_{2}}(y_{1},y_{2}) = C\left(F_{1}(y_{1}), F_{2}(y_{2}); \theta\right),  $$

generates a 2-variate joint distribution for the *Y*_*k*_’s, where *θ* is an association parameter. Hence, we can use paramet- ric families of copulas to generate a joint density $f_{Y_{1},Y_{2}}$ with marginal densities given by $f_{Y_{1}}$ and $f_{Y_{2}}$ [16]. The parameter of association may be difficult to interpret in some cases. To this end, the well-known Kendall’s *τ*∈[−1,1], a more interpretable measure of association, is a popular choice. It is defined to be the probability of concordance minus the probability of discordance between two independent random vectors [17]. For a deeper insight about copulas the reader is referred to [18] and [19].

### Mixed binary-continuous copulas

We are particularly interested in the case of building an inferential framework for two variables, one being continuous and the other one binary. However, a copula function is uniquely determined if and only if both random variables are continuous [16] limiting the direct applicability of copula to the discrete framework [20]. To circumvent this difficulty, we make use of the latent variable representation for binary regression models.

Let the random variable $\phantom {\dot {i}\!}Y_{2i} \sim F_{Y_{2i}}(.)$ be the continuous response of interest from each of *N* subjects and let $\phantom {\dot {i}\!}Y_{1i} \sim F_{Y_{1i}}(.)$ be the associated binary response indicator, *i*=1,…,*N*. For a particular realization (*y*_1*i*_,*y*_2*i*_),*y*_1*i*_ takes the value 1 when the corresponding *y*_2*i*_ is observed and a value 0, when *y*_2*i*_ is missing. These outcomes may arise in a breast screening program, for example, where a binary outcome indicates a woman that has yet reached the menopause and the continuous outcome may denote her age at menopause. Additionally, let $Y_{1i}^{*}$ be the unobserved continuous latent variable underlying *Y*_1*i*_, such that $Y_{1i} = \mathbbm{1}(Y_{1i}^{*} > 0)$, where *𝟙* is the indicator function. Without loss of generality, and for simplicity, we chose the cut-point at zero. This leads to a marginal logit model for the missingness indicator *Y*_1_, with the advantage of having a clear interpretation for the doctors who are familiarized with the interpretations on the log(*odds*) scale.

Together with the response variables, *Y*_1*i*_ and *Y*_2*i*_, a series of explanatory variables are also recorded and collected in an individual-specific vector ***v***_*i*_ containing, e.g., binary, categorical, continuous and spatial variables. The copula function contributes to build the joint distribution of the pair (*Y*_1*i*_,*Y*_2*i*_) given fully observed covariates of interest. Parametric models indexed by a vector of parameters ***β***, possibly including regression coefficients, will be considered to relate the covariates to the responses. The vector ***β***^⊤^ is partitioned as (***β***_1_,***β***_2_,*θ*) where ***β***_1_ and ***β***_2_ are for the marginal models of the missing mechanism and the continuous response, respectively. The joint distribution function $F_{Y_{1i}^{*},Y_{2i}}$ of $Y_{1i}^{*}$ and *Y*_2*i*_ is given by

2 $$ F_{Y_{1i}^{*},Y_{2i}}(y_{1i}^{*},y_{2i}) = C\left(F_{Y_{1i}^{*}}(y_{1i}^{*}), F_{Y_{2i}}(y_{2i}); \theta\right),   $$

with

3 $$ g_{1}(\mu_{Y_{1i}^{*}}) = \eta_{1i}(\boldsymbol{v}_{1i}; \boldsymbol{\beta}_{1}), \quad g_{2}(\mu_{Y_{2i}}) = \eta_{2i}(\boldsymbol{v}_{2i}; \boldsymbol{\beta}_{2})  $$

where *g*_1_(.) and *g*_2_(.) are link functions mapping the covariates to the marginal location parameters, $\mu _{Y_{1i}^{*}}$ and $\mu _{Y_{2i}}$, whose choice is governed by the parametric space. The linear predictors *η*_1*i*_(.;.) and *η*_2*i*_(.;.) depend on the outcome-specific covariate vectors, ***v***_1*i*_⊆***v***_*i*_ and ***v***_2*i*_⊆***v***_*i*_ and in the parameters, ***β***_1_ and ***β***_2_.

### The likelihood

In order to directly estimate the joint distribution of *Y*_1_ and *Y*_2_ as a bivariate copula, we need to consider that the joint distribution can be written as

4 $$\begin{array}{*{20}l} & P(Y_{1i} = y_{1i}, Y_{2i} \leq y_{2i})  \\ &= \left\{\begin{array}{ll} F_{Y_{1i}^{*},Y_{2i}}(0, y_{2i}), & y_{1i} = 0 \\ F_{Y_{2}}(y_{2i}) - F_{Y_{1i}^{*},Y_{2}}(0, y_{2i}), & y_{1i} =1 \end{array}\right. \quad \;  \\ &= \left\{\begin{array}{ll} C\left(F_{Y_{1i}^{*}}(0), F_{Y_{2i}}(y_{2i}); \theta \right), & y_{1i} = 0 \\ F_{Y_{2i}}(y_{2i}) - C\left(F_{Y_{1i}^{*}}(0), F_{Y_{2i}}(y_{2i}); \theta\right), & y_{1i} = 1 \end{array}\right..  \end{array} $$

From (4) we can write the joint density

5 $$\begin{array}{*{20}l}  & f_{Y_{1i},Y_{2i}}(y_{1i}, y_{2i})  \\ &= \left\{\begin{array}{ll} \frac{\partial C\left(F_{Y_{1i}^{*}}(0), F_{Y_{2i}}(y_{2i}); \theta \right)}{\partial F_{2i}(y_{2i})} \times f_{Y_{2i}}(y_{2i}), & y_{1i} = 0 \\ 1 - \frac{\partial C\left(F_{Y_{1i}^{*}}(0), F_{Y_{2i}}(y_{2i}); \theta \right)}{\partial F_{2i}(y_{2i})} \times f_{Y_{2i}}(y_{2i}), & y_{1i} = 1 \end{array}\right. \end{array} $$

The likelihood function for the parametric vector (***β***_1_,***β***_2_,*θ*) with data (*Y*_1_,*Y*_2_) may be represented as a combination of the likelihood function for individuals without missing responses, *Y*_1*i*_=1, and for individuals with the response missing, *Y*_1*i*_=0. Considering (5) and for now omitting the dependence on the outcome-specific covariates, we can write the likelihood for our copula model [10, 21]

6 $$\begin{array}{*{20}l} & \mathcal{L}(\boldsymbol{\beta}_{1}, \boldsymbol{\beta}_{2}; \theta)  \\ & = \prod_{i=1}^{N}\left[ P\left(Y_{1i}^{*} >0; \, \boldsymbol{\beta}_{1}\right)f\left(y_{2i} | y_{1i}^{*} > 0; \, \boldsymbol{\beta}_{2}, \theta\right) \right]^{y_{1i}}  \\ & \quad \times \left[P\left(Y_{1i}^{*} \leq 0; \, \boldsymbol{\beta}_{1}\right) \right]^{1-y_{1i}}  \\ & = \prod_{i=1}^{N} \left[ (1 - F_{Y_{1i}}(0 ; \boldsymbol{\beta}_{1}) f\left(y_{2i} | y_{1i}^{*} > 0; \, \boldsymbol{\beta}_{2}, \theta\right) \right]^{y_{1i}}  \\ & \quad \times \left[F_{Y_{1i}}(0 ; \boldsymbol{\beta}_{1})\right]^{1-y_{1i}},  \end{array} $$

where

7 $$ \begin{aligned} &f_{Y_{2i}|Y_{1i}^{*}}(y_{2i}| y_{1i}^{*} > 0)\\ &= f_{Y_{2i}|Y_{1i}}(y_{2i}| y_{1i} = 1)\\ &= \frac{\partial F_{Y_{2i}|Y_{1i}} \left(y_{2i} | y_{1i}=1 \right)}{\partial y_{2i}}\\ &= \frac{1}{ 1-F_{Y_{1i}}(0)} \frac{ \partial \left[ F_{Y_{2i}}(y_{2i}) - F_{Y_{1i},Y_{2i}}(0,y_{2i}) \right]}{\partial y_{2i}}\\ & = \frac{1}{ 1-F_{Y_{1i}}(0)} \left[ f_{Y_{2i}}(y_{2i}) - \frac{\partial F_{Y_{1i},Y_{2i}}(0,y_{2i})}{\partial y_{2i}}\right]\\ & = \frac{1}{ 1-F_{Y_{1i}}(0)} \left[ f_{Y_{2i}}(y_{2i}) - \frac{\partial C\left(F_{Y_{1i}}^{*}(0), F_{Y_{2i}}(y_{2i})\right) }{\partial y_{2i}}\right] \end{aligned}  $$

represents the density function of *Y*_2*i*_ given $Y_{1i}^{*}$. Note that we have not explicitly specified a model for $f_{Y_{2}|Y_{1}}$. It appears as the result of the marginal models chosen but mainly because of the copula function considered to capture the relation. If, instead of a MNAR assumption, we consider the data as MAR, then given $\boldsymbol {v}_{i}, Y_{1i}^{*}$ and *Y*_2*i*_ will be deemed conditionally independent, i.e.

8 $$ f_{Y_{2i}|Y_{1i}^{*}} (y_{2i} | y_{1i}^{*}; \boldsymbol{v}_{i}) = f_{Y_{2i}} (y_{2i} | \boldsymbol{v}_{2i}),   $$

and the likelihood (6) can be simplified.

## Imputation methodology

The primary goal of this work is to draw inferences about the distribution of *Y*_2_, representing the age at menopause, given a set of observed covariates, by considering the primary analysis model [*Y*_2_|***v***_*i*_]. The most popular approach would be to estimate the parameters of this distribution using only the observed values of *Y*_2_, yet estimates from such an analysis would be less efficient than they would be if we had observed *Y*_2_ for every individual. Recovering information via an imputation technique, e.g. multiple imputation (MI), should allow to retrieve some of the information about *Y*_2_ that is not available.

The underlying idea of MI is similar to prediction procedures, i.e. the observed data is used to predict plausible values but taking into account the uncertainty accrued from the imputation process. Those values are sampled from an adequate predictive distribution. To reflect the uncertainty attached to the procedure this process is repeated many times to obtain several complete sets of data, which are free of missing data [22]. A common misunderstanding about MI is that it is restricted to a MAR setting but the theory of MI is completely general and also applies to MNAR [23].

The work [24] warns about the typical naive approach of averaging the functionals of the distributions obtained according to each posterior predictive distribution. Instead, they advise to follow the approach in [25][pp. 159–162] that mixes the draws from the posterior predictive distributions from each completed data set and use those mixed draws to summarize the posterior quantities of interest. In particular, they find that the usual advice for MI with modest fractions of missing data which states that five or ten completed data sets are adequate for inferences can result in unreliable estimates. Additionally, the typical routine of estimating posterior quantiles in each completed data set and then averaging them across the data sets may produce unreliable estimates as well.

In the next two subsections we present two methodologies of imputation based on two different R packages that allow for multiple imputation under chosen work-models. Both are very flexible and the user is offered a variety of options for building the imputation model. This contrasts to most packages available that are often limited to simple models like the homoscedastic normal linear regression model [12].

### Imputing with a copula approach

This section introduces an imputation procedure, which is valid under the MNAR assumption, inside a bivariate copula approach considering a continuous response variable and a missing indicator. This procedure is easily implemented using the GJRM package in R.

Multiple imputation is a concept closely related to the Bayesian philosophy where the imputations are obtained by sampling from the posterior predictive distribution of the missing data given modelling assumptions and the observed data,

9 $$\begin{array}{*{20}l}  {}f(\mathcal{Y}_{\text{mis}} | \mathcal{Y}_{\text{obs}}, \boldsymbol{v}_{i}) = \int f(\mathcal{Y}_{\text{mis}} | \boldsymbol{\Phi}, \boldsymbol{v}_{i}) \, f(\boldsymbol{\Phi} | \mathcal{Y}_{\text{obs}}, \boldsymbol{v}_{i}) \; \mathrm{d}\boldsymbol{\Phi}, \end{array} $$

where, in our case, $\mathcal {Y}_{\text {obs}} = \left \{ y_{2i}: y_{1i}=1 \right \}$ and $\mathcal {Y}_{\text {mis}} = \left \{ y_{2i}: y_{1i}=0 \right \}, i=1,\ldots,N$; $f(\boldsymbol {\Phi } | \mathcal {Y}_{\text {obs}}, \boldsymbol {v}_{i})$ is the posterior distribution of all the parameters combined in the vector, ***Φ***. Unfortunately, the package GJRM does not support Bayesian inference, so samples of posterior distributions are not available. The posterior predictive distribution of the missing values is approximated by considering an approach based on the asymptotic normal approximation to the posterior distribution, $ f(\boldsymbol {\Phi } | \mathcal {Y}_{\text {obs}}, \boldsymbol {v}_{i})$, [11, 26], i.e. considering that $\boldsymbol {\Phi } \sim \mathcal {N}_{p}\left (\hat {\boldsymbol {\Phi }}, - \hat {\mathcal {H}}_{p}\right)$, where $\mathcal {H}_{p}$ is the model’s Hessian and $\hat {\boldsymbol {\Phi }}$ are the estimated parameters obtained by penalization of the likelihood in (6) [21]. After this, the imputation procedure is reduced to two steps: (i) draw $\tilde {\boldsymbol {\Phi }}$ from the multivariate normal $\mathcal {N}_{p}\left (\hat {\boldsymbol {\Phi }}, - \hat {\mathcal {H}}_{p}\right)$ and then (ii) draw a candidate $\tilde {y}$ from $ f\left (\mathcal {Y}_{\text {mis}} | \tilde {\boldsymbol {\Phi }}, \boldsymbol {v}_{i}\right)$ to replace the value not observed.

The package has the built-in function imputeSS(), which takes a fitted gjrm object and imputes the missing values. Although, the mixing of the “posterior imputed values” to which we allude above must be the carried on by the user. Additionally it does not provide an option to conduct imputations from a truncated distribution, which in our case would be extremely useful.

### Imputing with GAMLSS models

If we advocate that the missing ages at menopause are MAR, instead of MNAR, then the parametric vector, ***β***_1_, of the model $f_{Y_{1}}$ is separated from ***β***_2_, the parametric vector of $f_{Y_{2}}$. This implies that conditional on ***v***, the distribution of *Y*_2_ can be inferred considering only the units with *Y*_2*i*_ observed and with (*Y*_1*i*_=1), and then used to predict the missing observations of *Y*_2_.

In this section, where the missing mechanism is deemed ignorable, we describe how generalized additive models for location, scale and shape via the gamlss package in R [9] may be used for MI. As with GJRM, this package is not Bayesian-based, so we cannot rely on posterior predictive distributions. However, the package considers the bootstrap predictive distribution as an approximation to the posterior predictive distribution [27, 28]. This is achieved by approximating the Bayesian posterior distribution $f(\boldsymbol {\Phi } | \mathcal {Y}_{\text {obs}}, \boldsymbol {v}_{i})$ in (9) by $f\left (\tilde {\boldsymbol {\Phi }} | \hat {\boldsymbol {\Phi }} (\mathcal {Y}_{\text {obs}}, \boldsymbol {v}_{i})\right)$, which is the sampling distribution of the imputation parameters evaluated at the estimated values. The values $\tilde {\boldsymbol {\Phi }}$ are the possible values of the imputation model parameters, $\hat {\boldsymbol {\Phi }}(\mathcal {Y}_{\text {obs}})$ is an estimator of such model parameters. If there are variables fitted as non-linear functions, a penalization of the likelihood is used. This sampling distribution, $f\left (\tilde {\boldsymbol {\Phi }} | \hat {\boldsymbol {\Phi }}(\mathcal {Y}_{\text {obs}}, \boldsymbol {v}_{i})\right)$, is obtained by fitting the model to several bootstrap samples. The set of all parameters obtained constitutes the sampling distribution.

This imputation algorithm may be subject to some tailored constraints depending on the problem at hand. In this case, it makes little sense to impute a value for a missing age at menopause which is lower than the actual woman’s age. Thus a truncated distribution may be more suitable. The task may be accomplished by using the gamlss.tr package, which allows users to define truncated distributions in GAMLSS models. Unfortunately, within the package GJRM, we do not have such option.

In short, the procedure is very similar to the one presented for the GJRM package, i.e. we have to perform the following steps: (i) draw $\tilde {\boldsymbol {\Phi }}$ from their sampling distribution and then (ii) draw a candidate $\tilde {y}$ from the truncated $f\left (Y_{\text {mis}} | \tilde {\boldsymbol {\Phi }}, \boldsymbol {v}_{i}\right)$ to replace the value not observed. Again, we combine the estimates obtained from each analysed complete data set using the recommendations in [24]. A detailed description of the algorithm used for the imputation process is given in [12] and [29].

## Modelling the age at menopause in central Portugal

In this section, we will typify the models driving the missing data mechanism (when assuming that the data are MNAR) and the age at menopause by considering the very flexible framework of the structured additive regression (STAR) models [30].

### Semiparametric predictors

In a regression framing, potentially all distributional parameters involved may be related to additive predictors containing regression coefficients and observed covariates. The use of adequate link functions ensures the restrictions on the parametric space. However, in this work we will be modelling only the location parameters of the distributions concerned.

The great flexibility of both R packages GJRM and gamlss facilitates the choice of the functional form specifications for the missing and observed response models. In the case of the GJRM package, we want to simultane- ously model the underlying missing indicator, $Y_{1}^{*}$, and the response, *Y*_2_, as we are under an MNAR assumption. Both models will be linked with the introduction of a bivariate copula [8], conditional on some covariates. In the MAR scenario, we will use the gamlss package to model only *Y*_2_ before and after the imputations.

The linear predictors for the location parameters of the distributions considered for the marginal models, $Y_{1i}^{*}$ and *Y*_2*i*_, have a semiparametric additive structure according to:

10 $$ \eta_{ki} = \boldsymbol{\lambda}_{k}^{\top}\ddot{\boldsymbol{v}}_{ki} + \sum_{j=1}^{J_{k}}s_{kj}(v_{kji}), \quad k=1,2,   $$

where ***λ***_*k*_ is a design coefficients vector; the set of binary covariates, $\ddot {\boldsymbol {v}}_{ki}$, is a subset of the *p*_*k*_ dimensional set of covariates, i.e. $\ddot {\boldsymbol {v}}_{ki}\subseteq \boldsymbol {v}_{ki}=\left \{{v}_{k1i},\ldots, {v}_{kp_{k}i}\right \}$, and *s*_*kj*_(*v*_*kji*_) are *J*_*k*_ unknown smooth functions modelling the effects of the subset of continuous or spatial covariates, $\dot {\boldsymbol {v}}_{ki}=\left \{{v}_{k1i},\ldots, {v}_{kJ_{k}i}\right \}$, such that $\dot {\boldsymbol {v}}_{ki} \cap \ddot {\boldsymbol {v}}_{ki} = \emptyset $.

We take the binary observed covariates, pregnancy, anov and breastf for entering the model with linear effects. The effects of the continuous information such as birth, ipccap and menarche may be non-linear. Spatial information enclosed in muni, viewed as a Markov random field, will be taken into account in order to see how the age at menopause differs between regions.

In this scenario, the location parameters for the missingness and age at menopause distributions are specified as:

11 $$\begin{array}{*{20}l} \eta_{1i} &= \; \lambda_{10} + \lambda_{11}\times {\mathtt{pregnancy}}_{i} + \lambda_{12} \times {\mathtt{anov}}_{i}  \\ &\quad +\lambda_{13}\times {\mathtt{breastf}}_{i} \; + s_{11}({\mathtt{birth}}_{i}) \;  \\ &\quad+ s_{12}({\mathtt{ipccap}}_{i}) + s_{13}({\mathtt{menarche}}_{i}) \;  \\ &\quad+ s_{14}({\mathtt{muni}}_{i}),  \end{array} $$

12 $$\begin{array}{*{20}l} \eta_{2i} &= \; \lambda_{20} + \lambda_{21}\times {\mathtt{pregnancy}}_{i} + \lambda_{22} \times {\mathtt{anov}}_{i} \;  \\ & \quad+\lambda_{23}\times {\mathtt{breastf}}_{i} \; + s_{21}({\mathtt{birth}}_{i}) \;  \\ & \quad+ s_{22}({\mathtt{ipccap}}_{i}) + s_{23}({\mathtt{menarche}}_{i}) \;  \\ &\quad+ s_{24}({\mathtt{muni}}_{i}),  \end{array} $$

where *s*(.) refers to a non-linear effect, which can be a smooth function defined via splines in the case of continuous variables, or a Markov random field smoother in the case where the spatial information concerns a set of area labels like the case of *s*_*k*4_(muni_*i*_). More details are given in “Flexible effects” section below. The covariates used are considered to potentially influence the age at menopause according to some previous researches and expert opinion, as long as they were available in the data set.

In the case of an MNAR assumption, both the linear predictors (11) and (12) have to be taken into account, while in an MAR scenario only (12) is considered.

### Copula model

By specifying a bivariate copula with association parameter *θ* we build a joint model to glue the marginal models for $Y_{1}^{*}$ and *Y*_2_. Our framework investigates the adjustment of several copulas. The copula most supported by the Akaike Information criteria (AIC) and Bayesian Information criteria (BIC) was the Joe copula rotated by 270^o^ (Table 3), whose non-rotated version is defined as

13 $$\begin{array}{*{20}l} C_{J}(u_{1},u_{2};\theta) = & 1-\left[ (1-u_{1})^{\theta} + (1-u_{2})^{\theta} \; (1-u_{1})^{\theta} (1-u_{2})^{\theta}\right]^{\frac{1}{\theta}}, \end{array} $$

where $\phantom {\dot {i}\!}u_{1}=F_{Y_{1}^{*}}(y_{1}^{*})$ and $\phantom {\dot {i}\!}u_{2}=F_{Y_{2}}(y_{2})$, represent our marginal distribution functions (see [8] for further copula function choices), whereas the rotated version allows for the shifting of the tail dependence to one of the four corners of the unit square and can be obtained considering

14 $$ C_{J;270}(u_{1},u_{2};\theta) = u_{1} - C_{J}(u_{1},1 - u_{2};\theta).  $$

### Marginal models

#### Flexible effects

The terms in (10), (11) and (12) expressing a non-linear (flexible) effect for a continuous covariate will be considered by using a linear combination of spline basis functions [31], i.e.

15 $$ s_{kj}(v_{kji}) = \sum_{l=1}^{L_{kj}} \gamma_{kjl} B_{kjl}(v_{kji}) = \boldsymbol{\gamma}_{kj}^{\top}\boldsymbol{B}_{kj}(v_{kji}),   $$

where *L*_*kj*_ is the number of splines basis functions, $\boldsymbol {B}_{kj}(v_{kji}) = \left (B_{kj1}(v_{kji}), \ldots, B_{kjL_{kj}} (v_{kji}) \right)^{\top }$ is the *i*th vector of dimension *L*_*kj*_ evaluated at the observation *v*_*kji*_ and ***γ***_*kj*_ is the corresponding vector of coefficients. The basis functions, *B*_*kjl*_, are generally chosen based on convenience. We choose penalized splines as proposed by [31].

Considering (15), the linear predictors defined in (10) can be further simplified as

16 $$ \eta_{ki} = \boldsymbol{\lambda}_{k}^{\top}\ddot{\boldsymbol{v}}_{ki} + \boldsymbol{\gamma}_{k}^{\top} \mathbf{B}_{ki}, \quad k=1,2   $$

where $\boldsymbol {\gamma }_{k}^{\top } = \left (\boldsymbol {\gamma }_{k1}^{\top }, \ldots, \boldsymbol {\gamma }_{kL_{kj}}^{\top }\right)$ and $\mathbf {B}_{ki}^{\top } =  \left (\boldsymbol {B}_{k1}(v_{k1i})^{\top },  \ldots, \boldsymbol {B}_{kJ_{k}}(v_{kJ_{k}i})^{\top }\right)$. The writing can still be simplified if one considers $\mathbf {X}_{ki}^{\top } = \left (\ddot {\boldsymbol {v}}_{ki}^{\top }, \mathbf {B}_{ki}^{\top }\right)$ and $\boldsymbol {\varphi }_{k}^{\top } = \left (\boldsymbol {\lambda }_{k}^{\top }, \boldsymbol {\gamma }_{k}^{\top }\right)$, resulting in

17 $$ \eta_{ki} = \boldsymbol{\varphi}_{k}^{\top} \mathbf{X}_{ki}, \quad k=1,2.   $$

#### Spatial effects

The ages at menopause in the central region of Portugal may exhibit some spatial dependence, i.e., observations from neighbouring areas are expected to be more correlated than distant areas. In this regard it can be useful to inspect a spatial clustering in order to see if some latent characteristics of the response variable may arise. For instance, we may consider a simplification of the *s*_*k*4_(*v*_*k*4*i*_) function in (10) and write that *s*_*k*4_(muni_*i*_)=*ξ*_*km*_,*m*=1,…,78, where every municipality is assigned a specific regression coefficient giving us the level of some random quantity within the *m*th region. In case of a spatial variable, like muni, a simple Markov random field smoother [32] is sometimes appropriate. Indeed, the map displayed on Figs. 3 and 4 may be viewed as an irregular lattice.

A key concept for models dealing with spatial information is that of a adjacency (weights) matrix, ***W***, in our case with dimensions (78×78). We take it to be symmetric and of binary elements based on geographical contiguity; *w*_*st*_=1 if the areas (*A*_*s*_,*A*_*t*_) defined in $\mathbb {R}^{2}$ share common boundaries, perhaps a vertex, denoted *s*∼*t*; while *w*_*st*_=0 otherwise, denoted *s*≁*t*. This neighbourhood specification of first order implies that if *s* and *t* are geographically adjacent areas, *w*_*st*_=1, then their respective spatial effects are correlated, whereas spatial effects related to non-contiguous areal units are conditionally independent given the remaining spatial effects.

Typically, a penalty matrix is used to reduce the effective number of parameters that result from this highly parametric models. The objective is to have the elements of the 78-length vector of specific spatial effects of nearby regions, $\boldsymbol {\xi }_{k}^{\top } = (\xi _{k1}, \ldots,  \xi _{k78})$, not too different from each other. Generally, the penalty is based on the squared differences between the coefficients of all possible combinations of neighbourhood and given by ***K***=(***D***_*W*_−***W***), where ***D***_*W*_ is a diagonal matrix with each element of its diagonal being equal to the sum of each row of the matrix ***W*** (corresponding to the number of neighbours of each region). The matrix thus obtained, ***K***, keeps a structure of adjacency because their elements are only not zero when indicating a neighbourhood relation [33]. If one looks at the penalty from a Bayesian hierarchical perspective, the penalty can be viewed as being induced by an (improper) Gaussian prior, i.e. $\boldsymbol {\xi } \sim \mathcal {N}\left (\boldsymbol {0}, \tau \boldsymbol {K}^{-1}\right)$, where *τ* is a precision parameter. Thinking this way, ***ξ*** and the neighbourhood structure can be viewed as an (intrinsic) Gaussian Markov random field (GMRF) with variance matrix ***K***^−1^ [11]. The ages at menopause between regions are then assumed conditionally independent given these random effects. This approach is very popular in disease mapping [34].

#### Selected marginal distributions

As already stated above, we chose the logit model to regress the presence/absence of menopause age on the covariates and among the several distributions considered for the marginal age at menopause we found that the Gumbel provides the best fit (Table 2), whose distribution and density functions, parametrized accordingly to the gamlss package, are:

18 $$ {}F_{Y_{2}}(y_{2}) = e^{-e^{-z}}; \quad f_{Y_{2}}(y_{2}) = \frac{1}{\sigma} e^{- z - e^{-z}};\quad z=\frac{y_{2}-\mu}{\sigma}.  $$

**Table 2:** Selecting the best fitting marginal distribution for the age at menopause

Marginal	AIC	BIC
Gamma	1318295	1317297
Gumbel	**1263542**	**1264326**
LogNormal	1328666	1329256
Normal	1298449	1299285
Student’s t	1289426	1290270
Weibull	1265325	1266310

The parametrization is in terms of location, *μ* (the mode), and scale, *σ*, which is reproduced by the GJRM package. The mean is given by *μ*+*γσ* and the variance is *σ*^2^*π*^2^/6, where *γ*≈0.5772 is the Euler-Mascheroni constant.

## Results

In this section, we compare the results obtained by deleting the women without menopause (a CCA) to the results obtained after the data set has been completed with imputed values under both MNAR and MAR assumptions. Meanwhile, we will compare the results by means of the two R packages – GJRM and gamlss – in order to analyse the robustness of our findings.

### Model selection

Several variations of the models were tested in order to examine the robustness of the results to the different specifications. Selection procedures for the marginal distributions, namely the one for the continuous response and for the most suitable copula function were carried out using the AIC and/or BIC. Complementary to these measures, we considered a suitable residual analysis. Based on these criteria we selected a Gumbel distribution for the age at menopause, *Y*_2_ (Table 2).

Because the Gaussian copula allows for both positive and negative signs of dependence between the marginal distributions, we begin with it and then, based on the sign of the dependence, we consider alternative specifications consistent with this initial finding. In this case, the values for the Kendall’s tau (Table 4 - last row) is negative, −0.91, with an associated 95% confidence interval of (−0.913,−0.906), indicating that those women who are missing the menopause age, *Y*_1*i*_=0, are more likely to have their menopause at older ages. Thus, we only consider copulas consistent with this sign of dependence (Table 3). Based on these same model adequacy measures already reported, the preferred copula is the Joe copula with a rotation of 270^o^.

**Table 3:** Selecting the best fitting copula. The NA represent situations where the algorithm failed to converge

Copula	AIC	BIC
N	1394988	1397140
PL	1393386	1395308
C90	1391835	1393702
C270	1397586	1399744
J0	1401004	1403095
J90	NA	NA
J270	**1391834**	**1393701**
G90	NA	NA
G270	1393482	1395444

**Table 4:** Regression coefficients and standard errors for the binary variables. (^∗^*p*<0.05,^∗∗^*p*<0.01,^∗∗∗^*p*<0.001). Results are on the scale of the linear predictor

	CCA; gamlss; Gumbel margin	gamlss after imputations truncated Weibull	GJRM; no imputations; logit; Gumbel; Copula=J270	gamlss after imputations produced with gjrm: Gumbel margin Copula=J270
intercept	50.09 (0.03) ^∗∗∗^	51.09 (0.03) ^∗∗∗^	50.97 (0.03) ^∗∗∗^	51.59 (0.03) ^∗∗∗^
pregnancy	0.28 (0.04) ^∗∗∗^	0.19 (0.03) ^∗∗∗^	0.27 (0.04) ^∗∗∗^	0.27 (0.03) ^∗∗∗^
breastf	0.13 (0.02) ^∗∗∗^	0.15 (0.02) ^∗∗∗^	0.24 (0.02) ^∗∗∗^	0.20 (0.02) ^∗∗∗^
anov	0.25 (0.02) ^∗∗∗^	0.30 (0.02) ^∗∗∗^	0.40 (0.02) ^∗∗∗^	0.34 (0.02) ^∗∗∗^
$\sigma _{Y_{2}}$	4.04	4.01	4.25	4.23
*τ*	–	–	-0.91	–
*θ*	–	–	-20.8	–

It is worthwhile to note that a rotation of 270^o^ for the Joe copula means that the joint distribution is better described by an association structure where the variability associated to the likelihood of being missing is larger for the cases with higher menopause ages (for an intuition of this picture the reader is refereed to [21]).

### Estimated effects

**Fig. 6:**
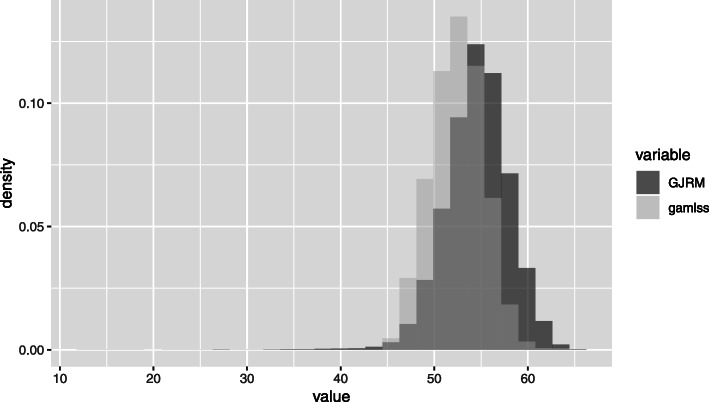
Two overlayed histograms showing one random sample of 20 imputations after applying the gamlss methodology considering a truncated Weibull distribution to impute the missing menopause ages (light grey) and after applying the copula approach (dark grey)

We considered 20 sets of imputed menopause ages which were then subject to a random sample to obtain our final imputed data set to be the subject of the analysis. This procedure is carried out twice (one for each missing mechanism considered). Thus, the results below for the data set completed with the imputations are based on such samples. Figure 6 shows the histogram for these two random samples obtained and consider: (i) an MAR scenario adjusted with the GAMLSS model (12) along with a truncated Weibull distribution to obtain the imputations accomplished within the package gamlss.tr; (ii) an MNAR scenario using the imputeSS function within the GJRM package. Although the shapes of the obtained distributions are similar, the distribution corresponding to the imputations via the imputeSS function is shifted towards larger values and has a larger lower tail. Based on the current knowledge of the biological menopause process, we can say that the imputations produced with the gamlss.tr package, which allows the user to use a truncated distribution for the imputations, in this case a Weibull, seem to be more in agreement with the values that are considered reasonable for a woman to reach the menopause age. Nevertheless, none of the imputed processes produced values above 67 years. The occurrence of menopause at the age of 69 and 70 is considered to be unrealistic [35].

Table 4 presents in 4 columns the estimates of the regression coefficients of the binary variables for 4 scenarios. In the first one, we consider a CCA within the gamlss package (before imputations); a Gumbel distribution for the age at menopause and the location parameter expressed according to (12) with an identity link function. Subsequently, we continue to consider the gamlss package but only after obtaining the imputations via the same package using a truncated Weibull distribution. The third scenario considers the copula approach according to “Modelling the age at menopause in central Portugal” section with a logit and Gumbel marginal models and a Joe copula rotated by 270^o^. The location parameters for the logit and Gumbel marginal models are expressed as in (11) and (12). The last scenario exposes the application of a GAMLSS approach to the completed data set obtained after the application of the imputeSS function in GJRM. From this table we can state that the different scenarios do not significantly differ in its estimates of the regression parameters for the binary variables. They are all significant and positive.

**Fig. 7:**
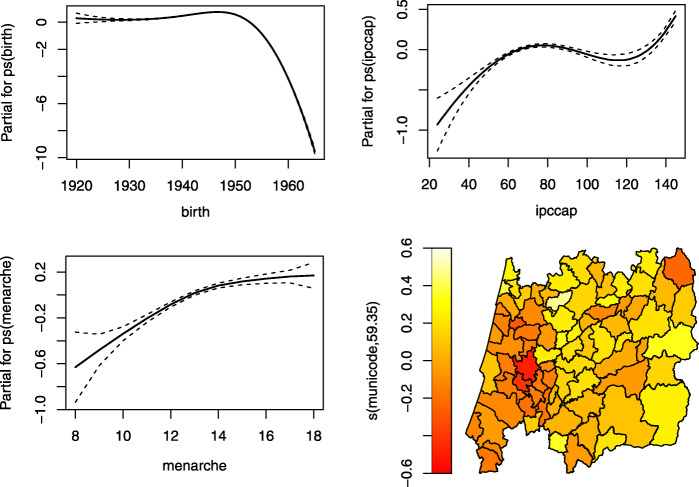
Results using gamlss to fit only the complete cases (CCA), i.e. without imputations. Results are plotted on the scale of the semiparametric predictor

**Fig. 8:**
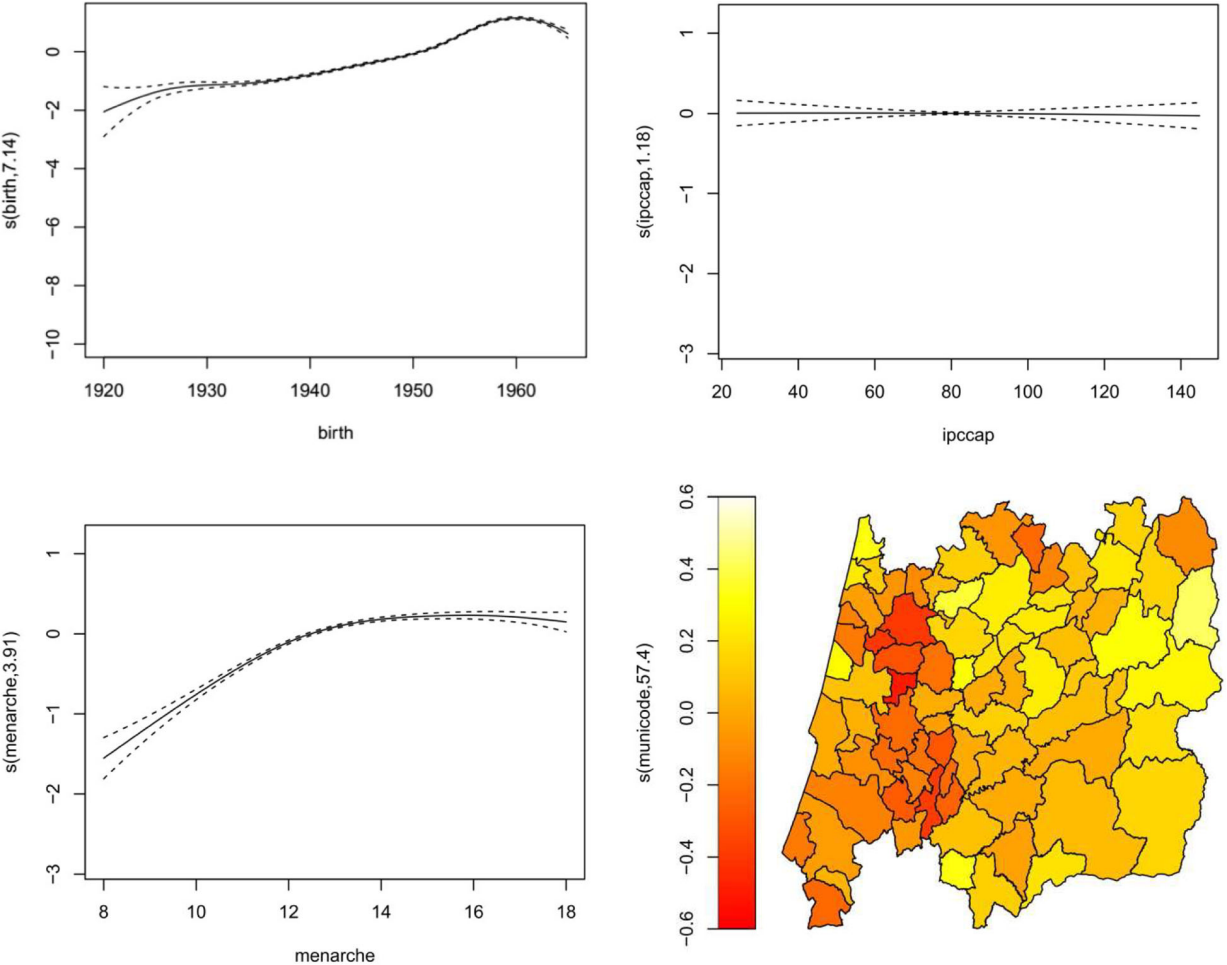
Results using gamlss to fit the completed cases, i.e. after the missing menopause ages have been replaced with the imputations considering a truncated Weibull distribution at the screening age to ensure that the imputed values are not lower than the actual woman’s age. Results are plotted on the scale of the semiparametric predictor

**Fig. 9:**
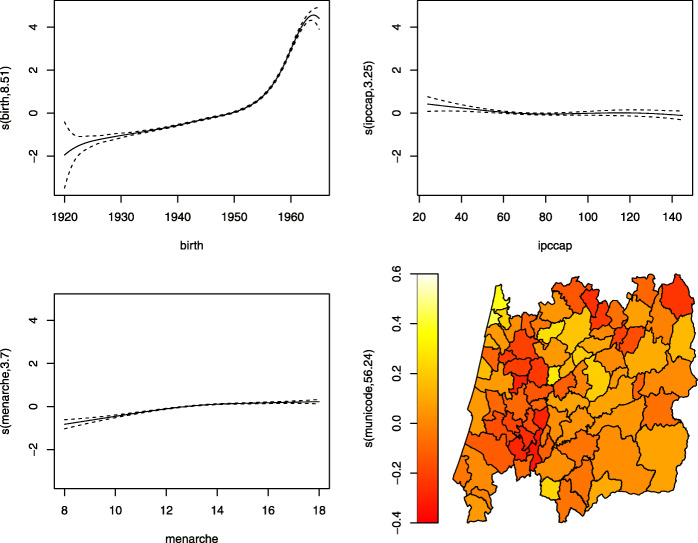
Results using GJRM to fit only the CCA, i.e. without imputations, considering a marginal Gumbel distribution for the menopause age and a Joe copula rotated 270^o^. Results are plotted on the scale of the semiparametric predictor

**Fig. 10:**
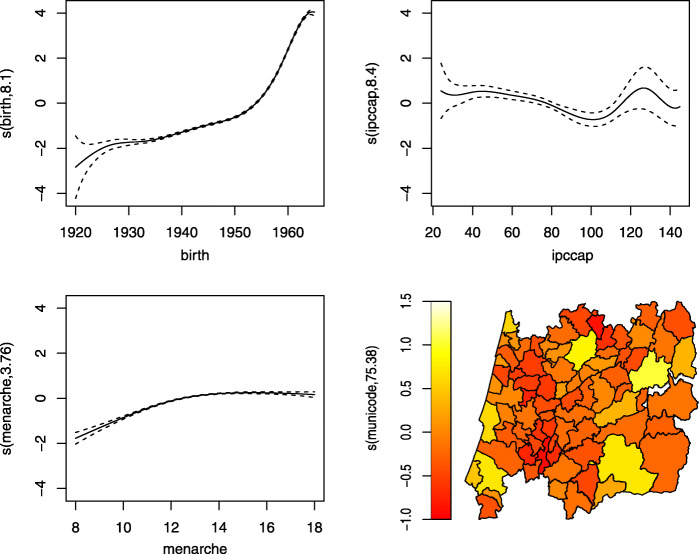
Results obtained with gamlss after filling up the missing values with one imputation using the copula approach. A marginal Gumbel for the menopause age and a Joe copula rotated 270^o^ were considered. Results are plotted on the scale of the semiparametric predictor

Figures 7, 8, 9 and 10 display the estimates of the non-linear effects for the continuous covariates in (12) for both MNAR and MAR assumptions. Figure 7 shows the results of fitting our model within the gamlss package before the imputations (corresponding to a CCA). The downward trend of the age at menopause when viewed as a function of the birth year is notorious, being in accordance with what had already been observed by [4]. Meaning that younger women are tendentiously having early menopauses. The variables ipccap and menarche have generally a positive relation with the menopause. Women living in municipalities with higher purchasing power tend to have late menopauses as well as women with late menarche. From the spatial clustering plot we might conclude that areas in the coast (Western) of Portugal tend to show early menopause.

A different story is told if one looks at Fig. 8, where the data has been completed with the imputations under an MAR assumption. The downward trend for younger women found in Fig. 7 born around 1950 is now reversed, implying that younger women now tend to have a late menopause. The effect of ipccap almost disappears and the menarche impacts negatively the menopause age only for those women that had their menarche until the age of 12. The spatial clustering remains more or less unchanged.

Figure 9 shows the results for the menopause age when fitting a copula model with the GJRM package under an MNAR statement. We noticed that this approach without imputations, i.e. using only the complete cases, already captures the “new” increasing behaviour of the birth variable (left top panel), that was observed in Fig. 8, despite the estimates of the parameters being slightly different (last two columns of the Table 4).

Figure 10 presents the results obtained with the gamlss package fitted to the data set after filling up the missing values with one imputation using the copula approach. Compared to Fig. 9, the variable that seems to be changing more its behaviour is ipccap. Those municipalities with a purchasing power slightly above the national average tend to show an increase in their menopause ages. The municipalities with higher ipccap are located in the coast of Portugal, and from the spatial plot (bottom right panel) those municipalities seem to have a negative spatial effect. Although these estimates may seem to point different conclusions, from our point of view we think that this is due to the spatial random effects showing that there is a need to incorporate new spatial information in the data because their confidence intervals do not contain zero.

**Fig. 11:**
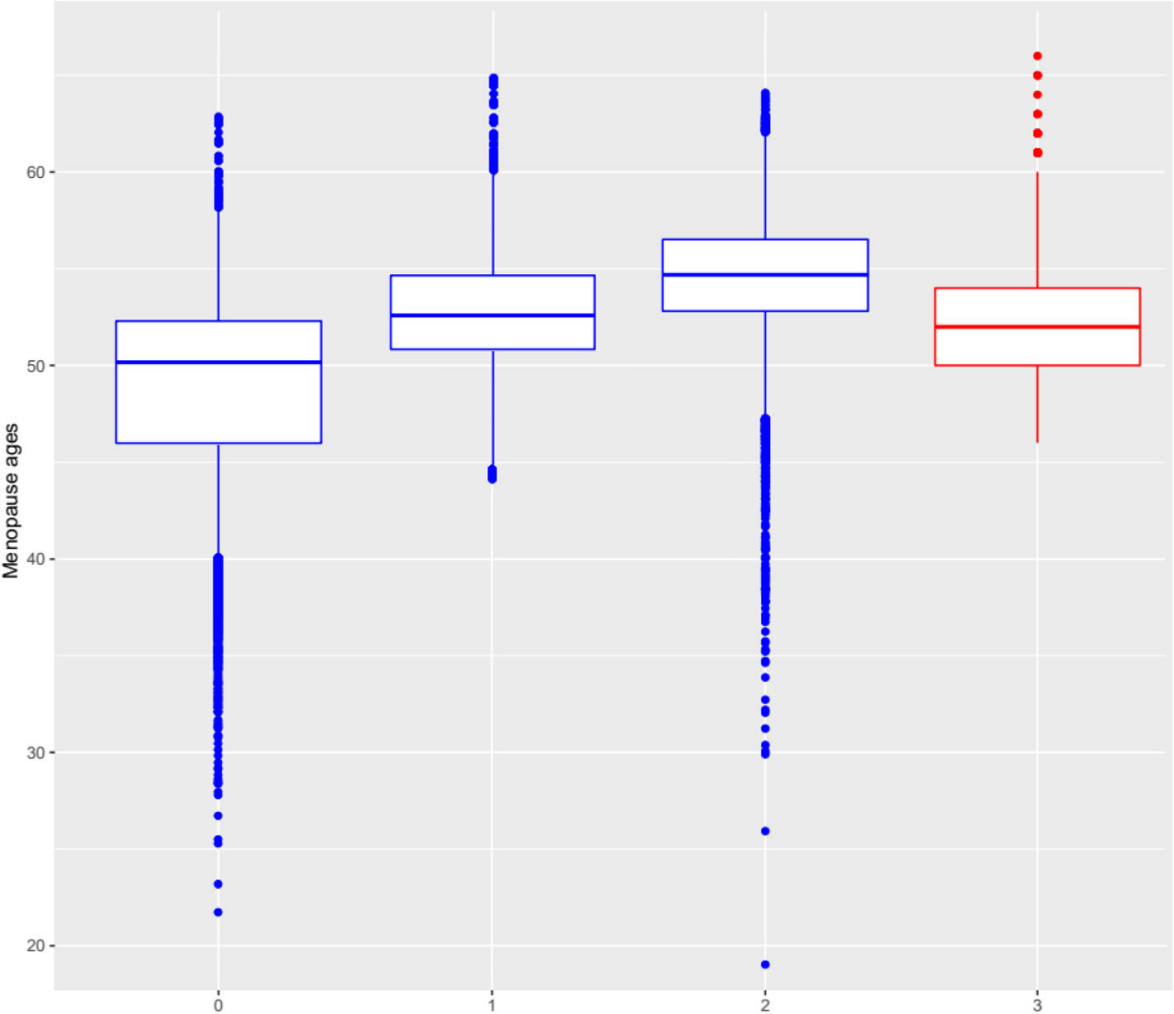
Boxplots for the menopause age considering only the set of women for whom menopause age was missing in 2010, but which was already observed in 2017: solely imputations without truncation in 2010 (0); solely imputations with a truncated Weibull in 2010 (1); solely imputations with a Copula approach in 2010 (2); true ages observed in 2017 (4)

Based on the validation analysis results shown in the Supplementary Material I and on Fig. 11 below, which compares the distribution of the age at menopause obtained in different scenarios of imputation, to the true observed ages in 2017, the MI approach using a truncated Weibull distribution within the gamlss package produces the best results, i.e., it produces complete data sets that are more in agreement with the reality than using the GJRM package that does not allow for truncation. Given that, and given the information provided by the Figs. 8 and 10, we can state that the age at menopause is increasing in the centre of Portugal. Younger women will, on average, experience the menopause a little later than women of previous generations.

Finally, we would like to emphasize that the first goal of this work was to assess the performance of some imputation procedures in retrieving the not yet observed ages at menopause. The decreasing behaviour of the menopause age as a function of the birth year, as in Fig. 7, is a fea- ture always present if we adopt a naive approach to the problem, i.e. if we do not input the not yet observed ages at menopause (see Supplementary Fig. S11 in the Supplementary Material I). If so, we will always be led to conclude that the age at menopause is decreasing at a very high rate for the younger generation. This is the main reason why we opt for only adjust models for the available information until 2010 and utilize the remaining one for performing an adequate analysis of the differences between the imputed values in 2010 and the true observed ones in 2017.

## Discussion

Missing data are often inevitable and many approaches have been considered to analyse data sets with these characteristics as alternatives to a complete case analysis. An imputation procedure for the missing ages at menopause is required if the study aims at analysing the trend of a variable in a setting that includes a cohort of women where the majority has already reached menopause and only a small part has not yet. This is always the case when we have a cohort whose age range includes the more likely age to reach the menopause. In settings, where either all women have already reached menopause, or neither woman is in menopause yet, there is no need to resort to any imputation procedure. From a statistical point of view, the first situation only requires the specification of an analysis model. The second situation cannot be inferred because we do not have information to predict individual menopause, unless we assume that they have the same characteristics as the older cohorts but then we would not be able to study the temporal trends across cohorts.

With a data set similar to the one that we worked with, not imputing the missing ages at menopause means that we will have to wait for all women belonging to the youngest cohorts to reach menopause in order to be able to assess the temporal trends of the menopause for a certain cohort of women. When fulfilling a data with imputations made in a proper way, we can model the temporal trends of the age at menopause immediately. This means that, in terms of public health, we will be studying the phenomenon of menopause without delays. The naive approach of simply delete the women without an observed menopause leads to biased results.

Our work presented two solutions for the problem of missing ages at menopause. One considering the data are MAR anf the other considering the data are MNAR.

Missingness at random is relatively easy to handle, and several pieces of software are already available for this task such as the R packages mice [36] or mi [37]. These procedures generally take as many variables as possible that might affect the probability of missingness to impute the missing values by specifying regression models without specifying a model for the probability of missingness. We tried both approaches but the results obtained were similar to the ones of a complete case analysis.

There is almost always a certain degree of dependence between the probability of missingness of the age at menopause and the values of the age at menopause itself. The question that can be asked is - how problematic is that dependence for our intentions? One thing that helps is to include as many predictors as possible in a model so that the MAR assumption is reasonable. This design can effectively transform MNAR data into MAR data, which is often used as a justification for assuming MAR. This strategy was followed with success in this work by adopting the imputation procedure, along with a truncated distribution for the menopause age, using the gamlss package. The other line of research that we pursued was to fit a joint model for the age at menopause and the probability of missingness. This was achieved using copulas which allowed us to model the situation with a non-ignorable missing mechanism.

With an unknown missingness mechanism, usually the relationship between the missingness pattern and the observations cannot be inferred from the data at hand. Therefore, an analysis assuming MAR should be accompanied by a sensitivity analysis as we did in the accompanying Supplementary Material II of this work. Since we also observed age at menopause in 2017 that we imputed in 2010, we checked the plausibility of the results and implicitly the underlying assumptions of the different methodologies by comparing the imputed to the true observed values for different models (see the Supplemen-tary Material I).

The drawbacks of our proposed approaches are: (i) computationally very demanding methods, particularly because we used spatial information and smoothing functions (P-splines) to model some of the functional relations; (ii) data set includes only a small subset of possible characteristics that can influence menopause age (e.g. we did not control for smoking status which is known to be an important factor). The aim of the paper was not to disentangle various risk factors for earlier menopause but rather to provide an imputation method in a screening setting with potentially a limited number of covariates and to emphasize how popular approaches might come to false conclusions when they do not adequately complete the generational cohort. (iii) When studying the age at menopause (a time-varying variable) one must be aware that period effects can potentially be mistaken for cohort effects because period, age and cohort effects are not easily separable if one wants to study the association with menopause age. Although we emphasize that the imputed values are not influenced by this relationship.

## Conclusion

In this work, we discussed two different approaches for dealing with missing menopause ages. One considers the data as MNAR and therefore we jointly model the missing data mechanism and the response variable of interest. The other approach considers an MAR data structure and thus only the statistical process of the age at menopause was modelled. Both are easy to understand and can be easily implemented using two packages (GJRM and gamlss, respectively) inside the popular R software.

Opting for the GJRM has the virtue of allowing the construction of a bivariate distribution in an easy and natural way by typifying a copula with a specific correlation parameter. After adjusting the model, the imputations are obtained via the imputeSS function. On the other hand, the imputation tools available within the gamlss are more useful because we are allowed to use truncated distributions while in the GJRM that feature is not available. This detail turns out to be decisive in the results obtained in the validation analysis presented in the Supplementary Material I. The differences between the imputed menopause values in 2010 and the true observed ages in 2017 are always smaller for the gamlss case.

## Supplementary Information

**Additional file 1** Supplementary Material I.

**Additional file 2** Supplementary Material II.

## Data Availability

The data sets used and/or analysed during the current study are available from the corresponding author on reasonable request.
