# Supplementary material for: Is age at menopause decreasing? – The consequences of not completing the generational cohort
Source: BMC Med Res Methodol. 2022 Jul 11;22:187. doi: 10.1186/s12874-022-01658-x (PMC9275053; doi:10.1186/s12874-022-01658-x)
Supplement: Supplementary file 1 — Additional file 1 Supplementary Material I. [file 12874_2022_1658_MOESM1_ESM.pdf]

Is age at menopause decreasing? – The consequences of not completing the generational cohort

(Supplementary Material I)

1 Validation Study

Conducting validity analyses to assess whether conclusions are robust when we are imputing multiple unobserved values is widely recommended. The database that we are working with is open, in the sense that it is constantly being updated with information from new women and women who are already part of it (longitudinal information). In 2017 we had been granted access to 20 130 women already screened in 2010 and who have since reached menopause. With these data in hands we can compare the imputed values for those women in 2010 with their real age at menopause, allowing us to check the reliability of the obtained results under the assumed missing mechanism.

2 gamlss

In what follows we present Table 1 with a summary of the differences between the observed and imputed menopause ages with a truncated Weibull distribution. Several graphical diagnostics comparing the observed and imputed data (**?**) are shown as well (Figures S1, S2 and S3).

	Min	Q_1	Median	Q_3	Max	95% CI (mean)
Solely observed ages (CCA) in 2010	20	47	50	52.81	65.68	$(49.09, 49.13)$
Observed and imputed ages in 2010	20	47	50	53	65.34	$(49.19, 49.23)$
True ages observed in 2017	46	50	52	53	65	$(51.66, 51.77)$
Solely imputed ages (gamlss)	28.06	52.68	52.30	56.68	65.67	$(54.56, 54.64)$
Differences: true value - imputation (gamlss)	-17.39	-5.18	-0.48	0.42	30.07	$(-2.41, -2.30)$
Solely imputed ages (gjrm)	28.06	52.68	54.60	56.68	65.67	$(54.56, 54.64)$
Differences: true value - imputation (gjrm)	-17.39	-5.18	-2.28	0.42	30.07	$(-2.41, -2.30)$

Table 1: Summary of the descriptive comparisons between observed and imputed menopause ages for a random sample of the imputed data set while considering the imputations via gamlss using a truncated Weibull distribution and the GJRM. Q_1 and Q_3 stand for the first and third quartiles, respectively. Last column represents the 95% confidence interval (CI) for the mean.

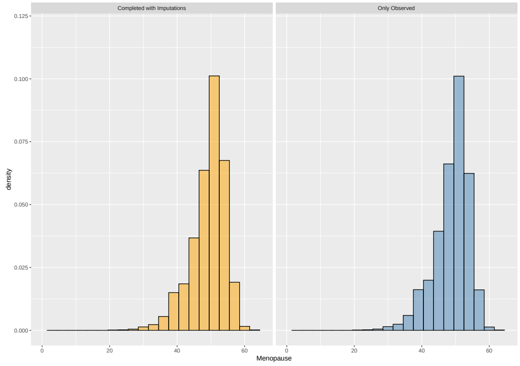

(a) Histograms of the completed menopause ages (left) and the observed ones until 2010 (right).

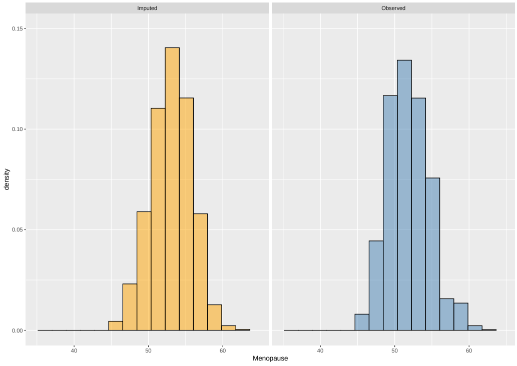

(b) Histograms of the imputed menopause ages until 2010 and the observed ones after 2010.

Figure S1: Graphics comparing the distribution of the completed, observed and imputed menopause ages.

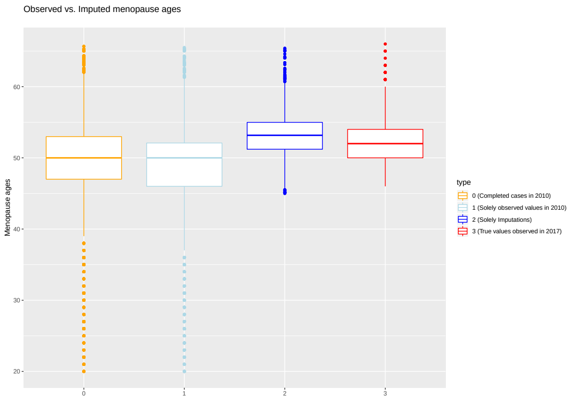

(a) Boxplots of the observed and imputed values

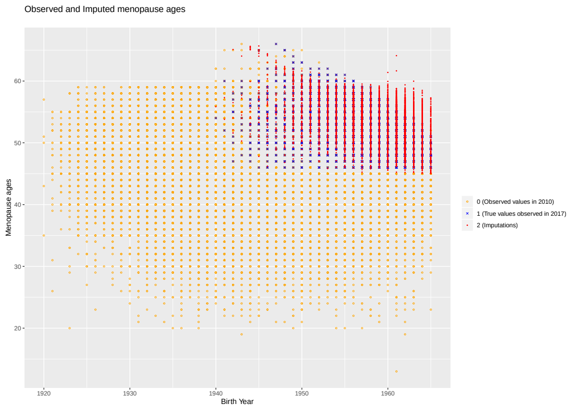

(b) Observed and imputed patterns.

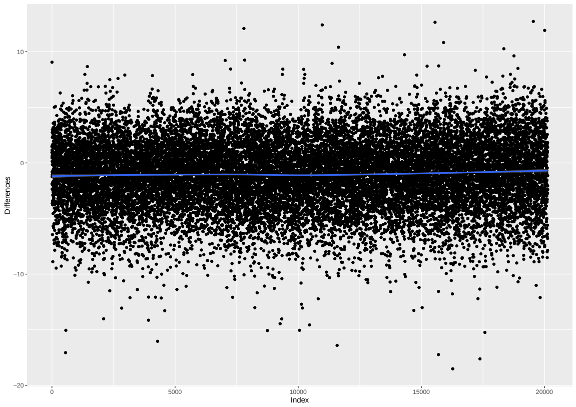

(c) Differences (Observed value - Imputed value) vs the index of the woman.

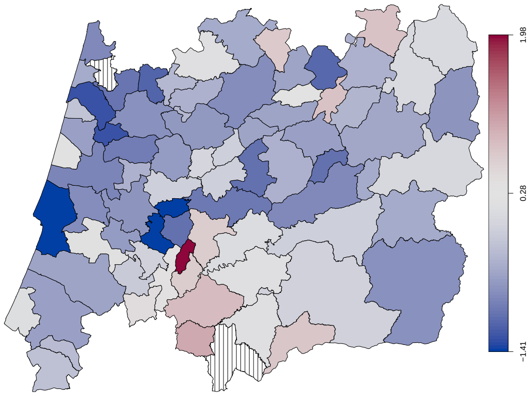

(d) Spatial pattern of the differences (Observed value - Imputed value). Two municipalities have no information available.

Figure S2: Graphics comparing patterns of the observed and imputed menopause ages.

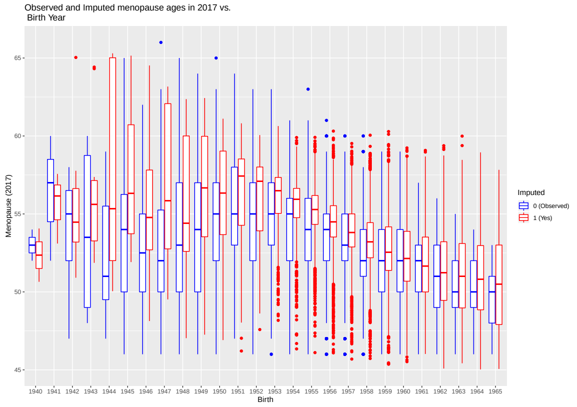

(a) Boxplots of the observed menopause ages in 2017 and the respective imputed values in 2010 vs. Birth Year.

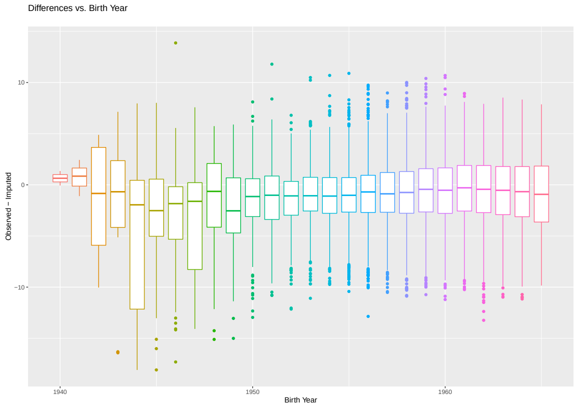

(b) Differences vs. Birth Year.

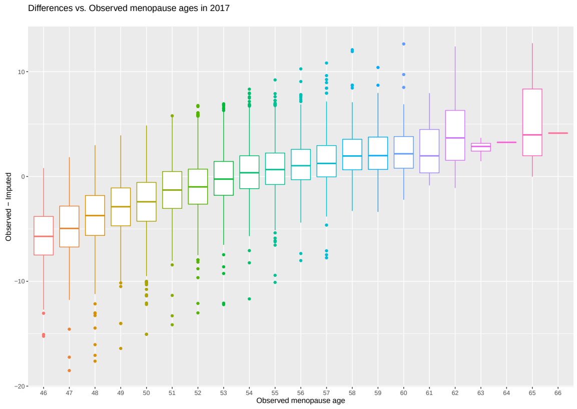

(c) Differences vs. Menopause age observed.

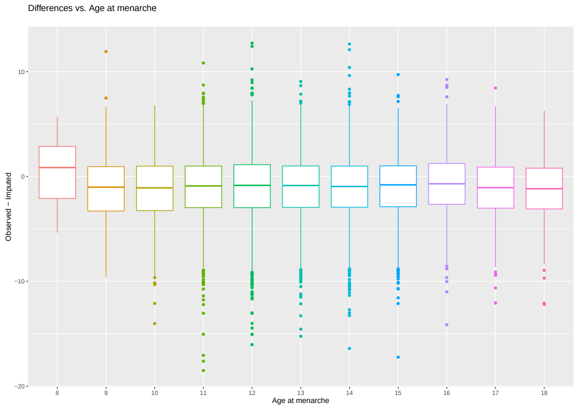

(d) Differences vs. Menarche age.

Figure S3: Boxplots of the differences against covariates.

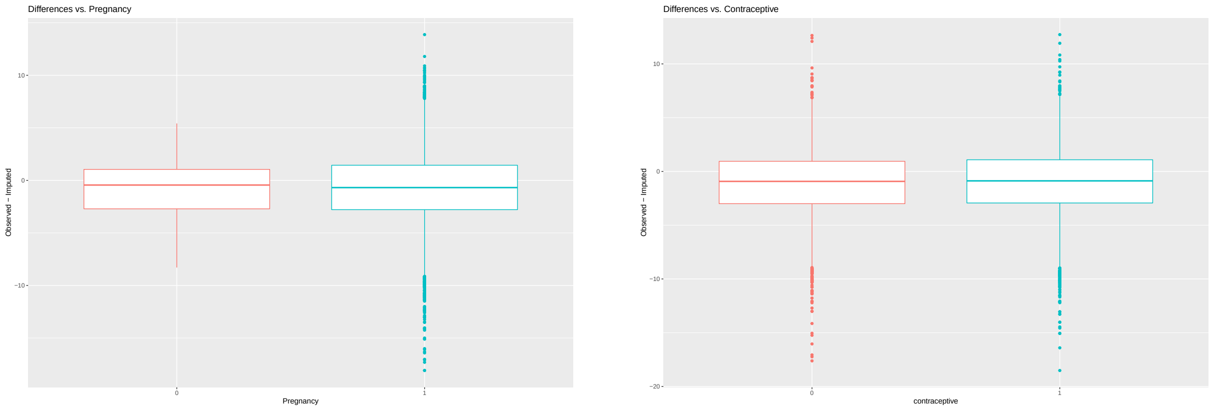

(a) Boxplots of the differences by Pregnancy.

(b) Boxplots of the differences by contraceptive.

Figure S4: Boxplots of the differences against covariates.

Concerning Table 1 rows show the summary for the following cases: i) only observed menopause ages being the unobserved discarded (Complete case analysis); ii) observed plus imputed menopause ages with a truncated Weibull distribution; iii) only the true menopause ages observed in 2017 for the cases with an imputed value in 2010; iv) solely the set of imputed menopause ages in 2010; v) comparison of the imputed ages in 2010 with the true observed values in 2017; they very similar (medians of 52.3 vs 52). The median difference is -0.48; vi) & vii) are the counterparts of cases iv) and v) for the case of imputations with a copula approach. In this case the median difference -2.8, thus yielding a worst performance.

Figure S1a shows that the distribution of the observations and the data completed with the imputations are both left skewed and very similar. Figure S1b highlights that the distribution of the true observed ages at menopause and the distribution of the imputations are quite similar, but obviously very different from the distributions in part a) of the figure, since here all ages are over 45 years old.

Figure S2 shows alternative comparisons of the observations' distribution and the imputed values, which were not readily clear from the former plots. Although, from both figures we can ensure that the distribution of the imputations is much closer to the true values observed for the menopause ages absent in 2010 than for those already observed in 2010. A clear sign that the imputations and the assumed model are capturing the behaviour of the missing ages at menopause.

An interesting plot is given in Figure S2b. The imputations for the menopause ages for the women born after 1958 are systematically above the observed values (disclaimer: these imputed values are for those women whose menopause age is not actually observed. A comparison between the value observed after 2010 and its value imputed in 2010 is given in Figure S2c). This is a rather interesting feature because the observed menopause ages for a woman born after this

year are again the lowest amongst the set of the menopause ages that we should expect for the women born from 1958 on. What this shows is not that the imputations are far from the observed true values, but that the imputation process is capable of capturing the global pattern of menopausal age, since if ones observe the pattern of imputations from that point on, we notice that they are in agreement with the pattern observed until that moment. This is due to the fact that women with the menopause observed and born after 1958 are those with the lowest ages of menopause. Later menopauses have not yet been observed. This pattern of the differences between imputations and expected values is what it is always expected to be observed for the younger women in this screening program. The fact that we have women born in 1940's for whom we had had not yet observed the age of menopause is mainly due to the abandonment of the screening program with the return happening only many years later.

Figure S2c shows that there is a global tendency for a small overestimation of the menopause ages. Finally, crossing S2d with the information in Figure 3 in the main text we see that municipalities with the largest missing rates of menopause are the ones with the largest predicted values (western coast). This is what should be expected, because if a value is missing that is because the woman is expected to have a latter menopause age. In this map it is also possible to see a municipality (Castanheira de Pêra) with the biggest differences. This is likely due to the fact that it is one of the municipalities with the smallest area in the central Portugal and also one of those with the lowest number of inhabitants, just over 3 000. In this case only 113 out of of 8 917 are from this municipality, representing 1.2% of the total.

Figures S3a and S3b show a set of boxplots concerning the differences between the observed and the imputed data for each year of birth. Again, some differences are revealed, including slightly higher median values in the observed data in the first years of this longitudinal study. Starting from 1944 this tendency is reversed. In Figure S3c, the differences have an increasing trend. At the lower end of the menopause ages, the imputation model overestimates the values, while at higher values there is a tendency for underestimation. This suggests that the imputation model has a poor predictive performance at the extremes of the menopause age distribution. Menarche does not seems to affect the errors (Figure S3d). The same is true for the covariates pregnancy and contraceptives (Figures S4a and S4b).

Finally we added Figure S5 where it is possible to observe what would happen with the imputations using this package within a MAR framework, but without truncating the imputation distribution. It is clear that the imputed age at menopause for a woman in 2010 would be much more in agreement with the age at menopause observed for other women in 2010, but much further from the true age of menopause for that woman observed in 2017. Thus the truncation plays a key role in our work.

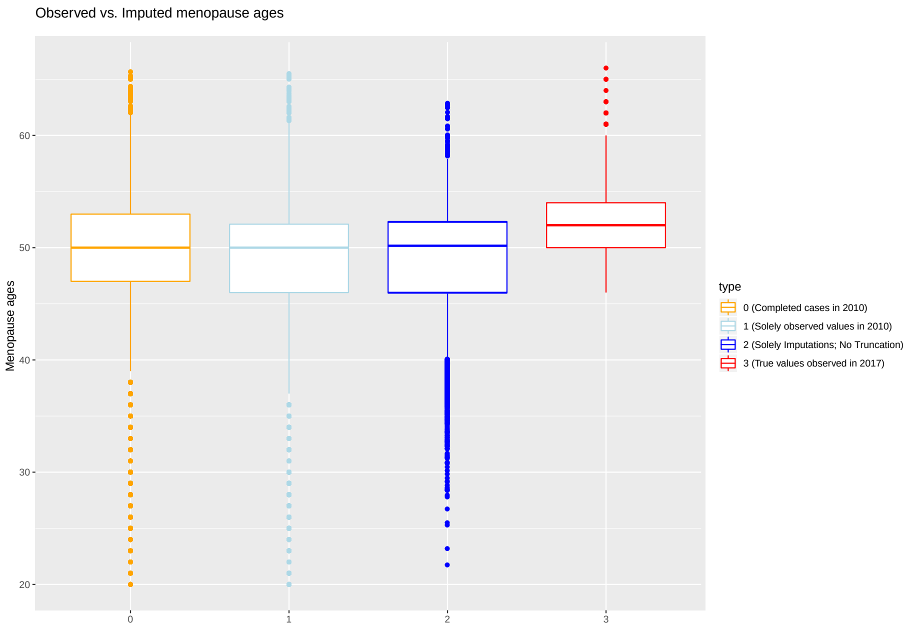

Figure S5: Boxplots of the observed and imputed values. The third boxplot represents the imputations using the gamlss but without considering a truncated distribution from which the imputations are sampled.

3 GJRM

It is worth recalling that the GJRM package does not allow the use of truncated probability distributions, thus some of the imputations for the menopause ages produced by this package have, as can be seen in the figures, predicted values lower than 45 years old.

In what follows we present a brief summary of the differences between the observed and imputed menopause ages while considering the imputations via GJRM package and several graphical diagnostics comparing the observed and imputed data (?) as shown in Figures S6, S7 and S8 below. As can be infered from the figures the difference between the imputed value in 2010 and the real value observed in 2017 is always larger with this approach compared to the ones obtained with the gamlss package with a truncated distribution.

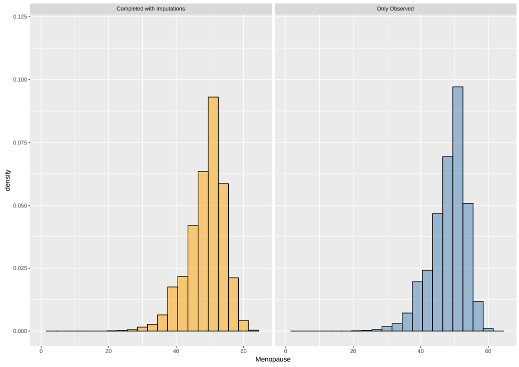

(a) Histograms of the completed menopause ages (left) and the observed ones until 2010 (right).

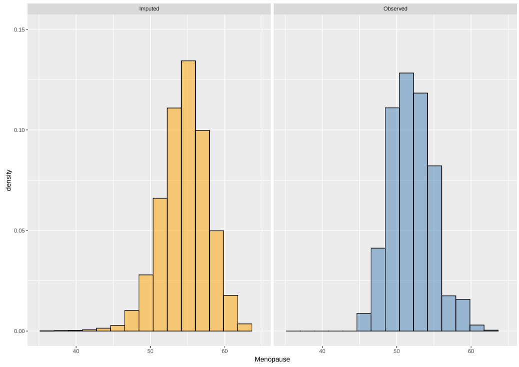

(b) Histograms of the imputed menopause ages until 2010 and the observed ones after 2010.

Figure S6: Comparing the distribution of the completed, observed and imputed menopause ages.

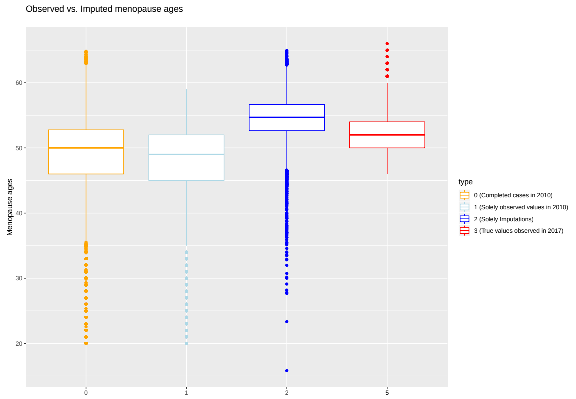

(a) Boxplots of the observed and imputed values

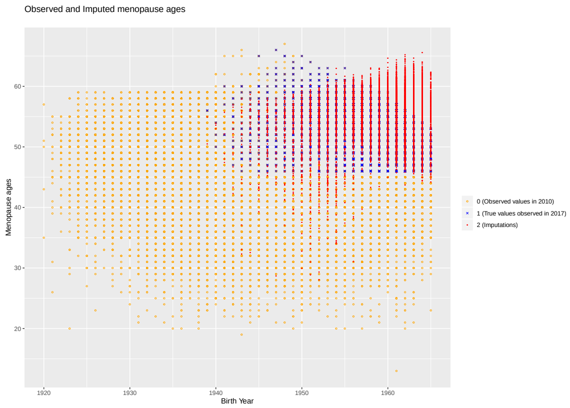

(b) Observed and imputed patterns.

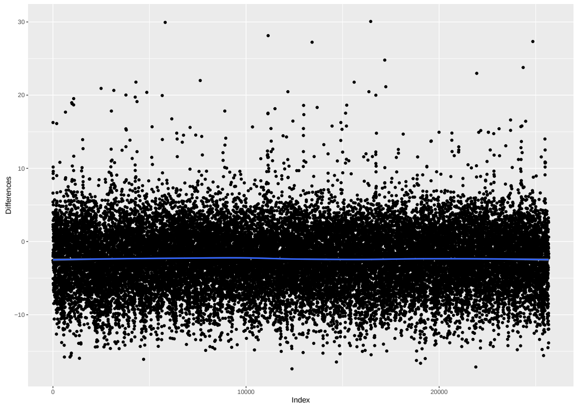

(c) Differences (Observed value - Imputed value) vs the index of the woman.

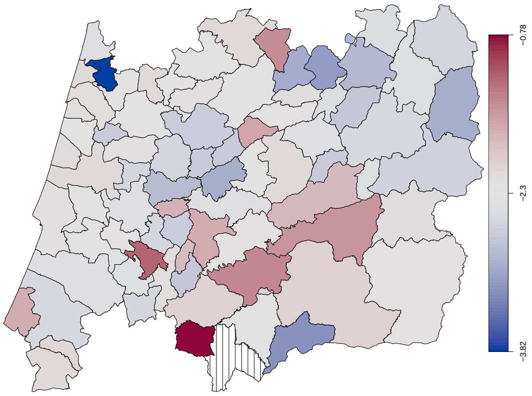

(d) Spatial pattern of the differences (Observed value - Imputed value). Two municipalities have no information available.

Figure S7: Comparing patterns of the observed and imputed menopause ages.

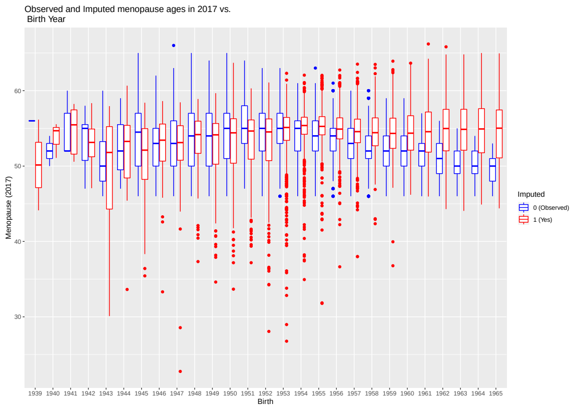

(a) Boxplots of the observed menopause ages in 2017 and the respective imputed values in 2010 vs. Birth Year.

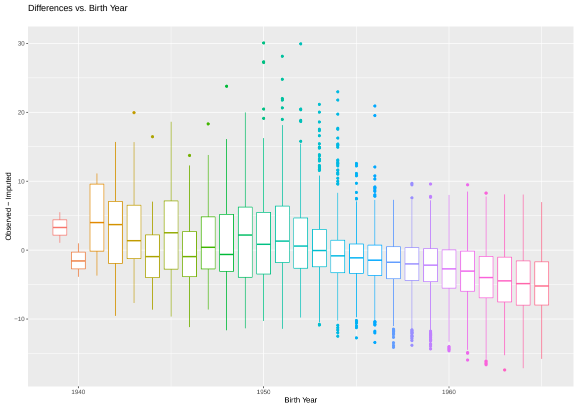

(b) Differences vs. Birth Year.

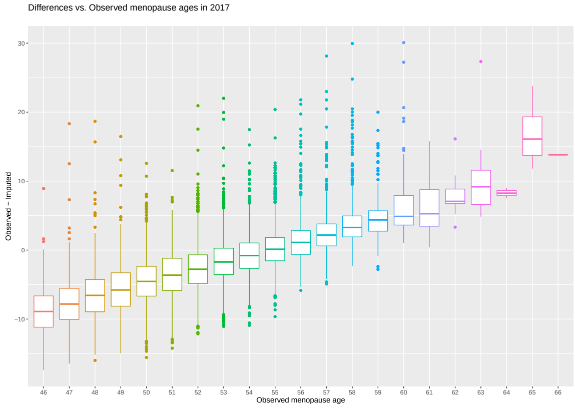

(c) Differences vs. Menopause age observed.

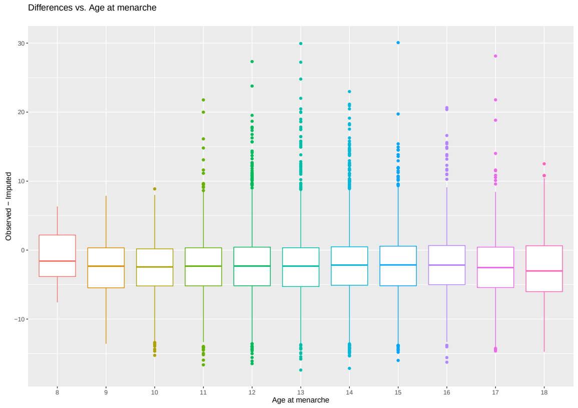

(d) Differences vs. Menarche age.

Figure S8: Boxplots of the differences against covariates.

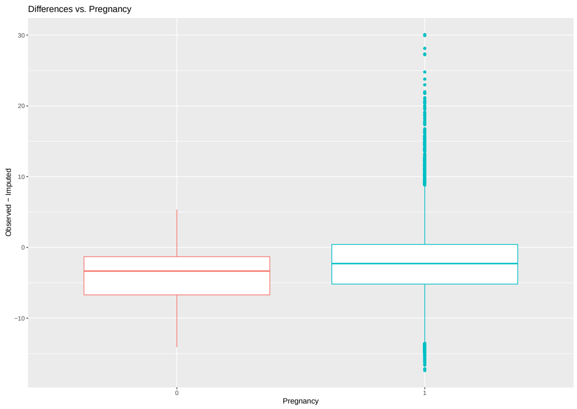

(a) Boxplots of the differences by Pregnancy.

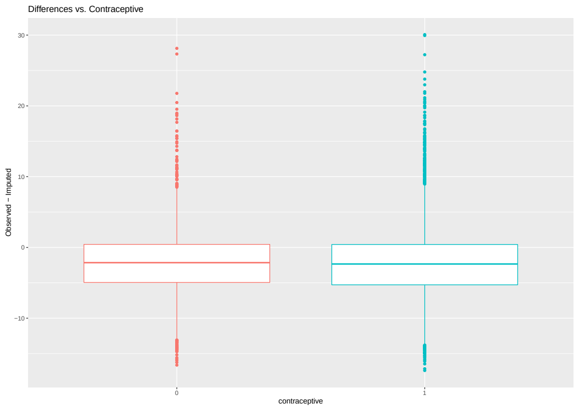

(b) Boxplots of the differences by contraceptive.

Figure S9: Boxplots of the differences against covariates.

4 Comparison of the adjustments

Considering the comments given in the precedent sections, here we will present only comparisons based on the gamlss package. The model considered for that adjustment is the same gamlss model that we used to produce Figure 8 in the main text, but instead of using the data completed with the imputations, we will only use the women that were in the database in 2010 and for which in 2017, the age of menopause was already known, i.e. we are considering women with a menopause age observed until 2010 and women in the dataset in 2010 but for whom the menopause was not yet observed, implying that these latter women have an age of menopause greater than 45 years old. In these conditions were 211 616 women. The idea is to see if the temporal behaviour of the covariates effects greatly differ, or not, from the plots in Figure 8 in the main text. In doing so we are comparing the reliability of the imputations, which are supposed to improve the model adjustment, with the estimates produced by a model applied to a dataset where all women had already reached the menopause. For example, the temporal effect of the variable `birth`, seems to roughly have the same shape in both figures, thus showing that our imputations are doing a good job in completing the missing information.

In Figure S11 we show the estimates for the smoothing terms for all the 311 539 women that we add access until 2017. Here we have approximately 33 000 women more than we had in the original dataset. The idea behind the construction of these plots is to see if the behaviour shown in Figure 7 in the main text for the birth year comes up again. And, in fact, that is what happens. So the steep downward slope of the curve after the year of birth 1948 seems to be a feature that we expect to have when the dataset is missing the ages of the menopause ages of the older women. When these women are included in the dataset (because they reached the menopause), as in Figure S10, this “strange” effect disappears. And this was already captured by the models fitted to the completed dataset with the imputations (Figure 8). These models have been able to shift the downward trend of the birth year effect approximately a decade, from 1948 to 1958.

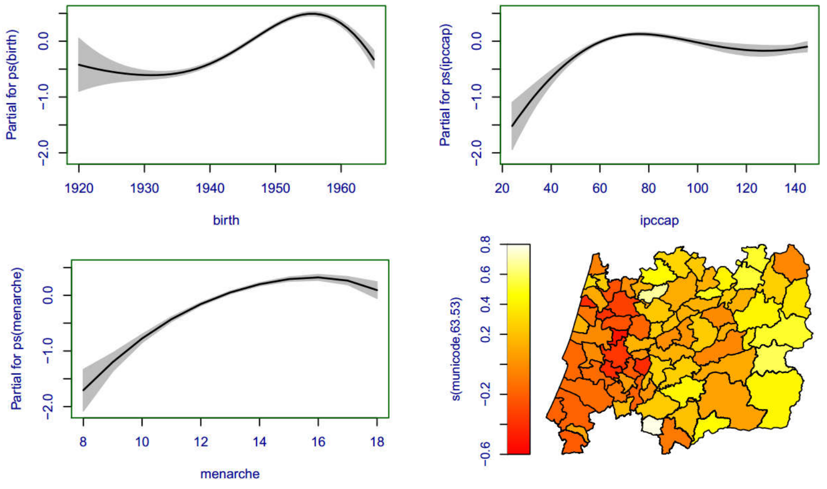

Figure S10: Results using the gamlss package to fit the age at menopause for women that were in the database in 2010 and for which in 2017, the age of menopause is already known. Results are plotted on the scale of the semiparametric predictor.

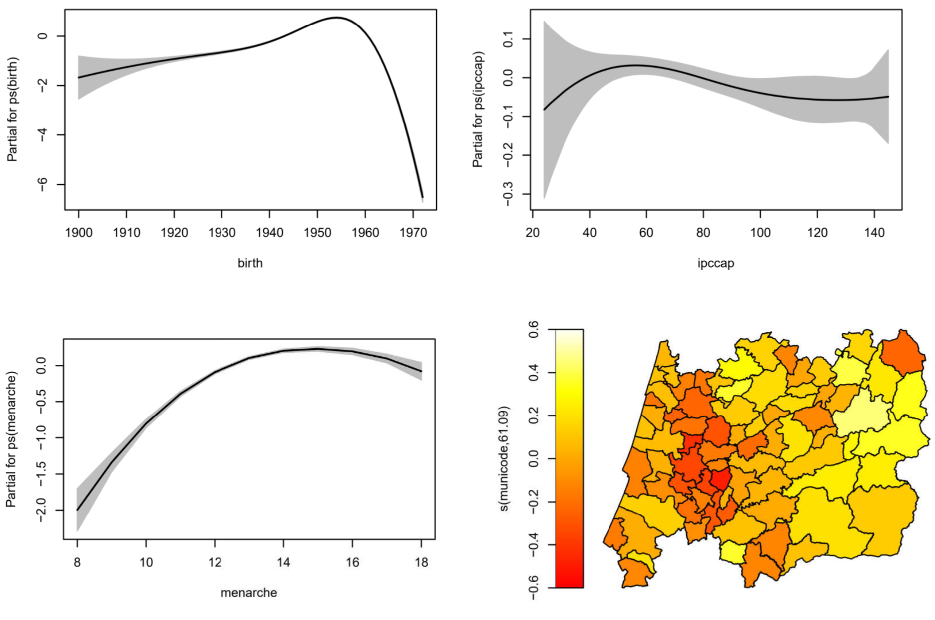

Figure S11: Results using the gamlss package to fit the age at menopause for women that were in the database in 2017 and for whom the age of menopause is known, irrespective of being or not in the dataset in 2010. Results are plotted on the scale of the semiparametric predictor.
