# Supplementary material for: Is age at menopause decreasing? – The consequences of not completing the generational cohort
Source: BMC Med Res Methodol. 2022 Jul 11;22:187. doi: 10.1186/s12874-022-01658-x (PMC9275053; doi:10.1186/s12874-022-01658-x)
Supplement: Supplementary file 2 — Additional file 2 Supplementary Material II. [file 12874_2022_1658_MOESM2_ESM.pdf]

Is age at menopause decreasing? – The consequences of not completing the generational cohort

(Supplementary Material II)

1 Sensitivity Analysis

We present a sensitivity analysis to study the influence of departures from the MAR assumption on the obtained estimates, thereby following the lines of other sensitivity analysis available in the literature, such as Buuren (2012); Moreno-Betancur and Chavance (2016); Leurent et al. (2018); Carreras et al. (2021).

One approach suggested in the literature includes adding a parameter that captures how the distribution of the missing ages at menopause could differ from the conditional distribution based on the observed menopause ages (Moreno-Betancur and Chavance, 2016; Leurent et al., 2018). This can be done, for example, by using an "offset" parameter, δ, representing the mean difference between the missing and observed ages. For instance, if we assume that women with a missing age at menopause are more likely to have higher ages at menopause then δ is positive, otherwise it is negative. The case without departures from the MAR is accounted for when $\delta = 0$. This parameter δ, allowed to vary across a set of plausible values, is the basis for assessing the sensitivity. It quantifies the degree of departure of the missing data distribution from the MAR model.

Thus, we explore the effect of artificially decreasing/increasing the multiple imputed age at menopause by subtracting/adding an amount of δ from the values imputed under the MAR assumption. This allows us to assess the robustness of an MAR analysis to departures from this assumption. We compare the results with a complete cases analysis in Table 1.

To obtain the imputed data under MNAR, we simply need to add to each MAR imputed value a δ value from a range of plausible/realistic values for this parameter and then assess how different assumptions could result in different estimates. The analysis is implemented in 3 steps similar to that in Leurent et al. (2018): (1) Perform multiple imputation considering a truncated Weibull distribution for the menopause ages; (2) Modify the imputed data to obtain the imputed data under MNAR by adding/subtracting to each MAR imputed value a plausible δ value; (3) Analyze the MNAR dataset considering eq. (12) within the `gamlss` function to obtain the estimates in Table 1 where $\delta \in \{-2, -1, 0, 1, 2\}$. These values were considered plausible as they represent the difference in menopause ages for the (possibly different) two groups – with and without an observed menopause age.

	**δ**				
	**MAR**	**MNAR**			
	**0**	**-2**	**-1**	**1**	**2**
**intercept**	51.09	50.0	50.7	52.3	52.1
**pregnancy**	0.19	0.41	0.33	0.38	0.38
**breastf**	0.15	0.18	0.54	0.24	0.27
**anov**	0.3	0.52	0.31	0.59	0.62

Table 1: Sensitivity analysis to the main coefficients of the model. First column coincides with the second column of the Table 4 in the main text.

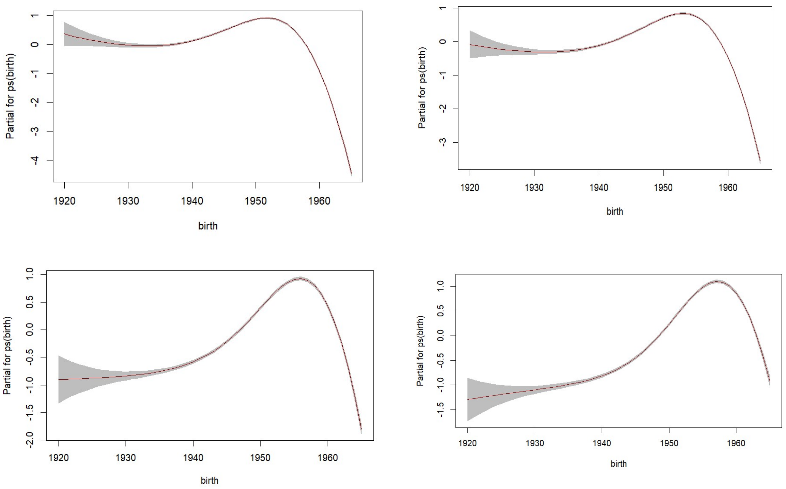

Figure S1: Results using gamlss to fit the completed cases after adding $\delta \in \{-2, -1, 1, 2\}$ to each imputed menopause age which were imputed from a truncated Weibull distribution at the screening age to ensure that the imputed values are not lower than the actual woman's age. Results are plotted on the scale of the semiparametric predictor.

It seems that the changes made to the imputed values (adding and subtracting small values) have little influence on the estimates of the coefficients for the binary variables (Table 1) which suggests that these results may not be influenced by departures from an MAR-mechanism or at least the missingness can be fairly explained by birth year and other covariates (i.e. MAR). Figure S1 shows the estimate for the non-linear effect of the birth year covariate where the data has been completed with the imputations under four scenarios of violations of the MAR assumption by adding $\delta \in \{-2, -1, 1, 2\}$ to each imputed menopause age, respectively. As can be seen from the figure, larger values of δ imply a shift of the graph to the right and down. We can interpret this as women born in the 30's and 40's always tend to have lower menopause ages irrespective of the δ adjustment made to the imputed ages.

We end this sensitivity analysis by stressing again that with our copula approach we shift the focus from the sensitivity analysis to the assumptions about the data distribution. This has the advantage of being

more readily implemented without worrying about assumptions on the missing mechanism type. In the paper we show both MAR and MNAR approaches but still lean towards MNAR.

References

Buuren, S. (2012). *Flexible imputation of missing data.* CRC Press, Boca Raton, FL. ISBN 9781439868249.

Carreras, G., Miccinesi, G., Wilcock, A., Preston, N., Nieboer, D., Deliens, L., Groenvold, M., Lunder, U., van der Heide, A., and Baccini, M. (2021). Missing not at random in end of life care studies: multiple imputation and sensitivity analysis on data from the ACTION study. *BMC Medical Research Methodology*, **21**(1). doi: 10.1186/s12874-020-01180-y.

Leurent, B., Gomes, M., Faria, R., Morris, S., Grieve, R., and Carpenter, J. (2018). Sensitivity analysis for not-at-random missing data in trial-based cost-effectiveness analysis: a tutorial. *PharmacoEconomics*, **36**(8), 889–901.

Moreno-Betancur, M. and Chavance, M. (2016). Sensitivity analysis of incomplete longitudinal data departing from the missing at random assumption: Methodology and application in a clinical trial with drop-outs. *Statistical Methods in Medical Research*, **25**(4), 1471–1489.
